# Digital interventions for subjective and objective social isolation among individuals with mental health conditions: a scoping review

**DOI:** 10.1186/s12888-022-03889-0

**Published:** 2022-05-12

**Authors:** Gigi Toh, Eiluned Pearce, John Vines, Sarah Ikhtabi, Mary Birken, Alexandra Pitman, Sonia Johnson

**Affiliations:** 1 Division of Psychiatry, Faculty of Brain Sciences, University College London, 149 Tottenham Court Road, London, W1W 7NF UK; 2 School of Informatics, University of Edinburgh, Crichton St, Newington, Edinburgh, UK; 3 Camden and Islington National Health Service Foundation Trust, St Pancras Hospital, St Pancras Way, London, NW1 0PE UK

**Keywords:** Social isolation, Loneliness, Digital interventions, Technology, Mental health conditions

## Abstract

**Background:**

Social isolation encompasses subjective and objective concepts. Both are associated with negative health consequences and are more prevalent among people with mental health problems than among the general population. To alleviate social isolation, digital interventions have potential as accessible alternatives or adjuncts to face-to-face interventions. This scoping review aimed to describe the types of digital interventions evaluated for feasibility, acceptability and effectiveness in alleviating social isolation among individuals with mental health problems, and to present an overview of the quantitative evidence yielded to inform future intervention design.

**Methods:**

We searched five electronic databases for quantitative and mixed methods studies published between January 2000 and July 2020. Studies were included if they evaluated digital interventions for individuals with mental health conditions, had subjective and/or objective social isolation as their primary outcome, or as one of their outcomes if no primary outcome was specified. Feasibility studies were included if feasibility outcomes were the primary outcomes and social isolation was among their secondary outcomes. A narrative synthesis was conducted to present our findings. The protocol was registered on Open Science Framework (doi:10.17605/OSF.IO/CNX8A).

**Results:**

Thirty-two studies were included for our review: 16 feasibility studies, seven single-group studies and nine effectiveness trials. There was great variation in the interventions, study designs and sample populations. Interventions included web-based programmes, phone-based programmes, blended interventions, socially assistive robots and virtual reality interventions. Many were feasibility studies, or otherwise not fully powered to detect an effect if one were present, thus preventing clear conclusions about clinical effectiveness. Satisfactory feasibility outcomes indicated potential for future trials to assess these interventions.

**Conclusion:**

Our scoping review identified a range of digital approaches utilized to alleviate social isolation among individuals with mental health disorders. Conclusions regarding clinical effectiveness cannot be reached due to variability of approaches and lack of large-scale randomized controlled trials. To make clear recommendations for digital social isolation interventions, future research needs to be based on rigorous methods and larger samples. Future studies should also focus on utilizing theory-driven approaches and improving existing approaches to advance the field.

**Supplementary Information:**

The online version contains supplementary material available at 10.1186/s12888-022-03889-0.

## Background

Social isolation can be defined as “a state in which the individual lacks a sense of belonging socially, lacks engagement with others, has a minimal number of social contacts and they are deficient in fulfilling and quality relationships” [1]. A distinction is often made between objective social isolation, where an individual has a small social network, or has infrequent contact with other people [2], and subjective social isolation, which is a perceived mismatch between an individual’s actual and desired social relationships, and which may include a perception of inadequate social support, leading to feelings of loneliness [3, 4]. Subjective and objective social isolation are conceptually distinct [5] and coincide to only a limited degree – it is possible to feel lonely while surrounded by many friends, but also to feel satisfied with one’s social life despite few social interactions [6].

Poor subjective and objective social isolation are associated with poor physical and mental ill-health [7]. Beyond cross-sectional findings that describe associations between subjective social isolation and cancer [8], depression symptom severity [9] and psychosis [10], longitudinal associations are also described between loneliness and the onset of depression [11]. Similarly, there is evidence to support cross-sectional associations between objective social isolation and poor health outcomes such as being diagnosed with “borderline personality disorder” [12], increased mortality rate [13] and higher risk of dementia [14]. In contrast, social support can be protective, being associated with better health outcomes such as improved immune function [15] and decreased likelihood of suicide attempts [16]. Greater perceived social support has also been found to buffer the rate and severity of psychological distress, depression, and anxiety [17, 18].

Compared to the general population, subjective and objective social isolation are found to be more prevalent among individuals with mental health disorders. Loneliness was more prevalent among people diagnosed with schizophrenia [19], while having fewer friends (i.e., smaller social network size) is also found to be more common among people with mental health problems, including people with adolescent-onset psychosis [20] and veterans with post-traumatic stress disorder (PTSD) [21]. Given these associations, the alleviation of social isolation is a potentially promising way of improving people’s mental health in both general and clinical populations. With the backdrop of the COVID-19 pandemic, the implementation of physical distancing rules to curb the spread of the virus has further heightened the need to understand both the impact of social isolation and how to reduce it effectively.

Despite the associations between social isolation and mental ill-health, interventions that target social isolation for people with mental health problems are still at the more preliminary stages of development [2, 22]. Many of these are in-person social interventions, which make use of peer support, increasing social and physical activities, interacting with animals and psychological therapies such as mindfulness-based and reminiscence therapies [23]. However, in part due to the longstanding stigma surrounding both mental health issues and loneliness, accessing in-person, face-to-face treatments for mental health-related issues can be difficult, whereas lack of resources and treatment delays also impede access [24]. Digital or technology-based approaches are thus potentially useful alternatives to face-to-face approaches, as digital literacy and usage are increasingly widespread, and individuals may feel more comfortable discussing sensitive and personal issues in the relative anonymity of an online context [25]. Well-developed digital interventions also have potential to be cost-effective [26], and to be scaled rapidly at low cost [27]. Twenty-four-hour availability is a further advantage, together with accessibility for people encountering barriers to help-seeking such as geographical location, disabilities or lockdown restrictions [28]. In relation to the issue of social isolation, digital technology such as social media and online communities have been shown to alleviate feelings of social isolation by facilitating feelings of social connectedness and reducing loneliness among young adults [29] and older adults [30]. Associations between greater internet use and increased social connectedness have also been demonstrated in older adults [31]. Thus, there is a growing body of research developing and assessing the use of digital interventions for social isolation. Providing an up-to-date appraisal of the extent and strength of this evidence is important given rapid technological advances and the possibilities for development and testing of novel tools.

Previous reviews of digital interventions for social isolation reviewed different types of digital interventions – some reviewed specific tools such as video calls [32], communication technologies [33], social robots [34] or computer and internet-training programmes [35], while some did not focus on any specific intervention and reviewed various forms of digital tools [36–38]. These reviews, however, focussed on subjective social isolation, older adults and/or general population samples. Only one review has examined the effects of computer- and internet-based training interventions on depression levels (in addition to loneliness) among older adults in the general population [35], but otherwise there has been relative neglect of populations diagnosed with mental health conditions.

While our initial intention was to conduct a systematic review, we decided to conduct a scoping review instead, as our preliminary scan of literature showed that potentially eligible studies used diverse study designs, involved clinical populations with a wide range of mental health conditions and used a variety of outcome measures. Compared to systematic reviews, scoping reviews are used to map the evidence in a field of study available to answer broader research questions [39], especially where ‘an area is complex or has not been reviewed comprehensively before’ [40]. In providing such a wide-ranging overview of the evidence, we aimed to identify studies on which future research and intervention development work might build. We widened our inclusion criteria beyond randomised controlled trials (RCTs) to other forms of experimental studies, including single-group studies and feasibility studies, as we anticipated few RCTs in an emerging research field addressing digital interventions for social isolation among people with mental health problems. The purpose of this scoping review was to stimulate future research in this digitalised age by mapping the available quantitative research evidence. We have included studies which yield data on the effectiveness of digital approaches in a mental health context, and papers that yield evidence on the feasibility and acceptability of studies on this topic as a potential prelude to investigate the effectiveness of the respective approaches. We pre-registered the review protocol with the Open Science Framework (OSF) online public database (doi:10.17605/OSF.IO/CNX8A).

## Methods

As our initial intention was to conduct a systematic review, our review protocol and inclusion/exclusion criteria were developed based on a systematic review framework. However, on commencing the systematic review, the studies retrieved were noted to have a high degree of heterogeneity, making it hard to answer our specific questions about intervention effectiveness and study feasibility. We therefore made a team decision to change our approach to that of a scoping review, restricted to quantitative studies, whilst using the same search criteria. Scoping reviews vary in the nature and types of evidence they include, depending on the research questions the review focuses on [41], as has been demonstrated in previous published reviews [42, 43]. We confined our review to include only quantitative studies, as this approach aligned with our objective of understanding the current state of evidence by examining quantitative work that has been done to date to assess the effectiveness of digital interventions for reducing social isolation, and the feasibility of carrying out such studies. Our review methodology aligns with a six-stage methodological framework adapted from Arksey and O’Malley [39] as well as Levac, Colquhoun and O’Brien [44]. We did not carry out the optional sixth stage that involves consulting stakeholders to validate study findings.

### Stage 1: identifying clear research question(s)

Our scoping review focused on three research questions: (i) for individuals with mental health problems, what are the types of digital interventions available to alleviate subjective and objective social isolation (encompassing concepts such as loneliness, perceived social support, social network size and social participation)? (ii) what have the research studies demonstrated about feasibility, acceptability and effectiveness of these interventions? and (iii) what type(s) of intervention(s) show promise for further testing in future large-scale trials?

### Stage 2: identifying relevant studies

#### Inclusion/exclusion criteria

To answer our research questions, we included studies that involved populations with mental health disorder(s) – this meant their eligibility criteria for recruitment included mental health conditions, assessed with diagnostic/symptom measures or diagnosed by mental health professionals, and including subsyndromal symptoms of mental health conditions. We included studies that either, within the context of social isolation, investigated the effectiveness of a digital intervention or assessed the feasibility of trialling the digital intervention in future large-scale studies. We included feasibility studies, where the primary aim is to assess whether and how future RCTs can or should be done [13], as well as RCTs, as this scoping review was aimed not only at assessing evidence of effectiveness, but also at exploring digital tools that are potential candidates for future research. We also included blended interventions (digital and face-to-face components) if the digital component of the intervention was delivered during 50% or more of the intervention period. No limits were placed on the forms of technologies or the scope of ‘digital’ approaches.

In terms of outcomes, studies were included if they reported outcomes related to subjective and/or objective social isolation as one of the primary outcomes, or as one of the outcomes in a study where the primary outcome was not specified. Studies that stated their main aim as investigating feasibility, acceptability or usability were also included if methods for the evaluation of the intervention’s clinical effectiveness regarding social isolation were being tested. For comprehensiveness we included studies measuring social isolation using validated outcome measures such as the Multidimensional Scale of Perceived Social Support (MSPSS), Lubben Social Network Scale (LSNS), single-item measures, and unvalidated outcome measures.

#### Search strategy

We included studies that were published from 01 January 2000 until July 2020 and used quantitative or mixed methods (extracting only quantitative data from mixed-methods studies). There was no restriction on the comparator or control condition. We excluded qualitative studies, case reports/series, reviews, meta-analyses and conference abstracts. Studies were excluded if the eligibility criteria required, alongside mental health conditions, a comorbid diagnosis of dementia, intellectual disability, autistic spectrum disorders, other neurological, organic or physical health problems.

Searches were conducted for relevant articles in five databases: Association for Computing Machinery Digital Library (ACM), Institute of Electrical and Electronics Engineers (IEEE) Xplore digital libraries, Embase, MEDLINE® and PsycINFO. The OVID interface was used to combine and search the latter three databases. Searches were limited to research carried out on humans. There were no language or geographical limitations to ensure a good representation of the target population by including all relevant studies. The complete list of search terms is provided in Supplementary file 1. Reference lists from all included studies were manually searched by the primary reviewer (GT). A second reviewer (SI) reviewed the reference lists from a randomly chosen 15% of the included studies, to check inter-rater reliability.

### Stage 3: selecting studies

Search results were imported into Endnote X9 software package for screening. GT reviewed all titles and abstracts from the retrieved articles and SI screened a random selection of titles and abstracts of 15% of the articles. All full-text articles for the candidate articles were then screened by GT using the agreed inclusion and exclusion criteria. At the same stage, SI randomly screened 15% of the candidate articles in full text. Any discrepancy regarding inclusion was resolved through discussion to reach consensus. When necessary, a third reviewer was involved to reach consensus.

### Stage 4: extracting and charting the data

Data extraction for included articles was conducted using a standardised proforma developed for the review, including publication year and country, participants’ demographics, sample size, study setting, study design, the nature of the intervention, details of follow-up, primary and secondary outcomes (including any feasibility outcomes), exclusion of participants, and the reasons for exclusion. GT carried out the data extraction of all articles and SI extracted data independently from a randomly chosen 15% of the included articles. Any discrepancy regarding data extraction was resolved through discussion and consensus, involving a third reviewer where necessary.

#### Quality assessment

Although quality assessment is not mandatory in scoping reviews, we conducted a quality assessment of included studies in order to explore strength of evidence in the current evidence base pertaining to our research questions, especially in studies where effectiveness was assessed. Following previous scoping reviews that also included quality assessment [45–50], we used the Mixed Methods Appraisal Tool (MMAT) [51] for quality assessment due to the inclusion of quantitative and mixed methods study in this review. We set the threshold for complete outcome data at 80% for this review, as per precedent in the literature [52]. Each domain was rated with ‘yes’, ‘no’ or ‘unclear’ in response to each statement. Each study was then rated to be at ‘high’, ‘moderate’ or ‘low’ risk of bias based on the number of domains that were fulfilled. Studies were rated as ‘low risk’ if four or five domains were fulfilled, ‘moderate’ if two or three were fulfilled, and ‘high’ if one or none of the domains were fulfilled. GT carried out quality assessment for all included articles, and SI randomly assessed 15% of the articles. Any discrepancy between the two reviewers was resolved through discussion and consensus, involving a third reviewer if necessary. Studies with low methodological quality were not excluded from the final synthesis, apart from those that failed to meet the two MMAT screening criteria (whether there are clear research questions, and whether the collected data address the research questions).

### Stage 5: collating, summarizing and reporting findings

As recommended in scoping review methodology frameworks, extracted data were collated and summarized into a descriptive and narrative summary of study characteristics [39, 44]. Comparisons across studies were made relating to their design and methodology, target populations with different mental health conditions, as well as characteristics and components of the interventions that were investigated. Using a thematic approach, we then classified the interventions evaluated in the included studies into groups to explore the nature of the interventions, feasibility to be conducted in future trials and potential effectiveness of the interventions [53]. To facilitate comparisons, we also presented the studies’ characteristics, intervention characteristics and findings in tables (Tables 1, 2, 3, 4, 5, 6 and 7).

**Table 1:** The study characteristics of feasibility studies, stratified by study design

**Feasibility/Pilot randomized controlled trial(s) (*n*** **= 3)**^a^							
**Author, year, country**	**Participants**	**Setting**	**Aim**	**Control/comparator condition**	**Follow up**	**Outcome measure(s)**	
Rotondi, 2005, [54•] USA	- Persons with schizophrenia (PWS): aged 14 or older and diagnosed with schizophrenia or schizoaffective disorder - Support persons: aged 18 or older	In- and outpatient psychiatric care units and rehabilitation centres	To assess the feasibility and preliminary efficacy of providing a psychoeducational program to participants’ homes via the Internet.	Intervention vs. treatment as usual (TAU) control	Long-term follow up: 3-and 6-month post-baseline	- Social isolation outcomes (for PWS and support persons): perceived social support via Perceived Support Scale (PSS) and Medical Outcomes Study Social Support Survey (MOS-SSS) - Feasibility outcomes: helpfulness, value, understandability and ease of use via Website Evaluation Instrument (WEI) - Other outcomes: perceived stress	
O’Mahen, 2014, [55•] UK	Mothers with postnatal depression symptoms and had given birth to a live baby last year	Netmums website and newsletter	To (1) establish recruitment and trial adherence rates; (2) determine treatment adherence and predictors of modules and telephone sessions; (3) assess the preliminary effectiveness of NetmumsHWD on depressive and anxious symptoms, work and social impairment, perceived support, and maternal self-reported bonding with her infant in order to help inform future sample size calculations; and (4) gather data on health care utilization at baseline and at post-treatment in preparation for a health economic assessment.	Intervention vs. TAU control	- End of treatment: 17-week - Long-term follow up: 6-month	Social isolation outcomes: perceived availability of support via Social Provision Scale (SPS) - Feasibility outcomes: treatment adherence and attrition - Other outcomes: depression, anxiety, work and social impairment, postnatal bonding and health service utilization \	
Hanssen, 2020, [56•] Netherlands	Adults (aged 18–60 years) with a schizophrenia spectrum disorders diagnosis	Community mental health treatment teams, hospitals, iand patient- and relative associations	To find out whether using an interactive smartphone application is feasible in a schizophrenia sample and whether providing personalised ESM-derived feedback can ameliorate symptoms and improve social functioning.	SMARTapp provided feedback according to the participants’ daily ESM entries vs. SMARTapp included only ESM questionnaires without personalised feedback.	End of treatment: 3-week	- Social isolation outcomes: loneliness measured by daily ESM questionnaire - Feasibility outcomes: usage and evaluation of SMARTapp - Other outcomes: positive and negative psychotic symptoms, social functioning, intelligence	
**Feasibility/Pilot non-randomized trial(s) (*n*** **= 1)**^b^							
**Author, year, country**	**Participants**	**Setting**	**Aim**	**Control/comparator condition**	**Follow up**	**Outcome measure(s)**	
Pfeiffer, 2017, [57•] USA	Individuals with an inpatient diagnosis of depression	VA Ann Arbor Healthcare System	To assess the acceptability and feasibility of enhanced social support with automated telephone monitoring for patients with depression discharged from psychiatric inpatient stays.	Social support provided by a family/friend VS social support provided by a trained peer specialist	- Mid-term follow up: 3-months - End of treatment: 6-month	- Social isolation outcomes: perceived social support via Multidimensional Scale of Perceived Social Support (MSPSS) - Feasibility outcomes: acceptability and satisfaction with the program - Other outcomes: depression severity, depression symptoms, anxiety, suicidal ideation, functioning, hopelessness, antidepressant adherence, readmission to inpatient psychiatry, outpatient mental health visits	
**Feasibility/Pilot single-group study(s) (*n*** **= 7)**^c^							
**Author, year, country**	**Participants**	**Setting**	**Aim**	**Follow up**		**Outcome measure(s)**	
Van Voorhees, 2005, [58•] USA	Adolescents (ages 18–24) with at least one risk factor for developing depression	Primary care settings in the USA (urban primary care and university health clinic)	To evaluate the acceptability, potential adverse effects, and potential benefits (i.e., reduction of risk factors for depression) of the intervention.	Time elapsed from enrolment to follow-up interview (completers) ranged from 1 to 6 weeks		- Social isolation outcomes: social support questionnaire-short form (SSQ-6) - Feasibility outcomes: acceptability (performance, satisfaction ratings) - Other outcomes: adverse effects, depressive symptoms, dysfunctional thinking	
Rice, 2020, [59•] Australia	Young people (aged 12–25 years) with “probable” social phobia	Headspace early intervention centres in north-western Melbourne, Australia	To examine the acceptability, feasibility, safety and potential clinical benefit (according to both clinical and social measures) of a novel digital intervention for young people with social anxiety. In addition to Entourage demonstrating acceptability, feasibility and safety, the intervention was expected to be associated with improved clinical (e.g., social anxiety, mood, wellbeing) and social functioning (e.g., social support, connectedness) outcomes between baseline and post-treatment.	End of treatment: 12-week		- Social isolation outcomes: social support network via LSNS, social connectedness via Abbreviated Duke Social Support Index (DSSI), Revised University of California, Los Angeles Loneliness Scale (UCLA-LS), Social Connectedness Scale (SCS) and Interpersonal Needs Questionnaire (INQ) - Feasibility outcomes: acceptability (participants’ use of the system), feasibility (capacity to recruit and retain young people in Entourage) and safety (monitoring the occurrence of adverse events and symptom deterioration) - Other outcomes: depression, wellbeing, social anxiety, self-esteem, emotion regulation, self-compassion, experiential avoidance, public speaking cognitions, guilt and shame.	
Rice, 2018, [60•] Australia	Young people (aged 15–24 years) with diagnosis of Major Depressive Disorder (MDD); either in partial remission or full remission	Early intervention clinics in Melbourne, Australia	To evaluate the acceptability, feasibility, usability and safety of an innovative moderated online social therapy (MOST) for depression relapse prevention in young people.	- End of treatment: 12-week		- Social isolation outcomes: social connectedness via MOS-SSS, social support via the 2-Way Social Support Scale - Feasibility outcomes: acceptability (user experience via Website Analysis and Measurement Inventory), feasibility, usability (frequency ratings and patterns of use) - Other outcomes: MDD diagnoses, depression symptom rating, strengths use, worry and anxiety	
Price, 2014, [61•] USA	Individuals (aged younger than 55 years) who presented for an injury that would satisfy a diagnosis criterion of PTSD	Recovery ward of a Level 1 Trauma Center	To determine the proportion of trauma patients that would consent to receiving daily text messages assessing mental health, determine response rates to daily text messages among trauma patients, identify predictors of higher rates of responding, assess patient satisfaction, and determine provider burden.	Long-term follow up: 1-month, 3-month		- Social isolation outcomes: MOS-SSS - Feasibility outcomes: satisfaction with the intervention - Other outcomes: depressive symptoms, PTSD symptoms, illness intrusiveness rating Scale, injury severity score.	
Ludwig, 2020, [62•] USA	Individuals (aged 18–35 years) with schizophrenia spectrum disorder and with a maximum of 5 lifetime years of treatment with antipsychotic medication	- Recruited from First Episode Psychosis clinics in North Carolina.	To examine the feasibility and acceptability of Horyzons for American clients receiving care at three FEP clinics in North Carolina.	- Mid-term follow up: 6-week - End of treatment: 12-week - Long-term follow up: 16-week		- Social isolation outcomes: loneliness via UCLA-LS, perceived social support and relationship quality via Social Provisions Scale (SPS) - Feasibility outcomes: usage (site logins) and satisfaction (perceived benefits or challenges of intervention) - Other outcomes: well-being, positive and negative emotions, subjective self-worth, psychosis-related symptoms, depressive symptoms, social/ occupational functioning	
Bailey, 2020, [63•] Australia	Young people (aged 16–25 years) with suicidal intent	Tertiary-level outpatient mental health service	To (1) evaluate the safety, feasibility, and acceptability of a MOST intervention (“Affinity”) among a sample of young people who were receiving treatment for major depressive disorder and had also experienced past-four-week suicidal ideation, (2) explore changes in cognitive and interpersonal targets of the Affinity intervention, as well as changes in self-reported depression and suicidal ideation.	End of treatment: 8-week		- Social isolation outcomes: Social Connectedness Scale-Revised (SCS-R) - Feasibility outcomes: safety, feasibility, acceptability - Other outcomes: suicidal ideation, depressive symptoms, perceived burdensomeness and thwarted belongingness, mindful attention awareness, self-compassion, problem solving, suicide severity rating, self-harm risk	
Alvarez-Jimenez, 2018, [64•] Australia	Young people (aged 15–25 years) of ultra-high risk (UHR) for psychosis	Specialised UHR research clinic (the PACE clinic)	To conduct an initial evaluation of MOMENTUM regarding its acceptability, safety and potential to improve social functioning in UHR young people	- Baseline and 2-month follow up		- Social isolation outcomes: perceived social isolation and social support via SPS and University of California, Los Angeles Loneliness Scale (UCLA-LS-3) - Feasibility outcomes: feasibility, acceptance (participants’ usage) and safety (adverse events) - Other outcomes: social functioning, subjective well-being, self-efficacy self-esteem, depressed mood, perceived stress, mindfulness, strengths use	
**Feasibility/Pilot mixed-methods study(s) (*n*** **= 5)**							
**Author, year, country**	**Participants**	**Setting**	**Aim**	**Control group**	**Follow up**	**Data collection method**	
						**Quantitative: outcome measure(s)**	**Qualitative**
Lim, 2020, [65•] Australia	Young people (aged 16–25 years) with diagnosis of psychotic disorder	Early psychosis services in Melbourne, Australia	To (1) develop a pilot digital smartphone application to target loneliness that is feasible and acceptable to young people with early psychosis, (2) develop an app that is usable to young people as one necessary step to reducing the likelihood of poor engagement, a common problem with mental health smartphone apps	None	- End of treatment: after participants complete at least 33 days of +Connect (T2) - Long-term follow up: 3-month post-treatment (T3)	- Social isolation outcomes: loneliness via Revised UCLA-LS - Feasibility outcomes: acceptability, feasibility, usability (content helpfulness, satisfaction ratings, completion rates, etc) - Other outcomes: social interaction anxiety, psychological well-being, positive and negative symptoms, depressive symptoms	- Semi-structured qualitative interview at T2 regarding participants’ experiences using the app
Lim, 2019, [66•] Australia	- Young people (aged 18–23 years) with Social Anxiety Disorder (SAD) - Young people (aged 18–23 years) with no diagnosable mental health disorder (recruited as control participants	- SAD participants: Local youth health service - Control participants: Australian university	To examine the acceptability, feasibility, and safety of +Connect in young people with or without social anxiety disorder.	SAD participants vs. control participants	- End of treatment: after participants complete at least 33 days of +Connect (T2) - Long-term follow up: 3-month post-treatment (T3)	- Social isolation outcomes: loneliness via Revised UCLA-LS - Feasibility outcomes: acceptability, feasibility, usability (content helpfulness, satisfaction ratings, completion rates, etc) - Other outcomes: social interaction anxiety, depressive symptoms	Semi-structured qualitative interview at T2 regarding participants’ experiences using the app
Gjerdingen, 2013, [67•] USA	Mothers aged above 16 years with postpartum depression	St. Paul hospitals, local practices, mothering Web sites and mid-wife referrals	To pilot a peer support intervention trial for mothers with postpartum depressive symptoms. Specifically, to test the feasibility of recruiting postpartum doulas, peer telephone supporters, and depressed mothers for a trial, and began to evaluate the impact of postpartum doula and peer telephone support as adjunctive treatments for depressive symptoms.	Postpartum doula vs. peer telephone support vs. no treatment control	- End of treatment: 3-month - Long-term follow up: 6-month	- Social isolation outcomes: social support via a 7-item scale: available Support, Importance of Support and satisfaction with support - Other outcomes: mental health history, health status	- Open-ended questions or opportunities for making further comments.
Dow, 2008, [68•] Australia	Individuals (aged 65 years or above) with mild depression, provide personal care for a co-resident relative, live in the Pyrenees subregion	Local newspapers, word of mouth and carer support groups	To test the feasibility of a computer intervention for promoting the health of rural carers in Australia.	None	Long-term follow up: 3-month	- Social isolation outcomes: loneliness via UCLA-LS - Other outcomes: depressive symptoms, carer burden	- Semi-structured interview regarding participants’ experiences using the computer and intentions regarding future use. - Group discussion about their experiences of the computer intervention
Campbell, 2019, [69•] Australia	Young people (aged 13–25 years) with mild-to-moderate depression or anxiety; contacting or previously engaged with Kids Helpline (KHL) for counselling; seeking support for issues related to family discord	Kids Helpline counselling service and Webchat	To develop the evidence base to validate the proof of concept for KHL Circles: a purpose-built, private, and secure SNS, designed to provide 24/7 group counseling to young people in Australia experiencing family discord.	None	- Mid-term follow up: 2-, 4- and 6-week - End of treatment: 8-week	- Social isolation outcomes: MSPSS - Other outcomes: depressive symptoms, anxiety, self-esteem,	Mid-term and final surveys asked for indications of helpfulness, whether participants would return to KHL Circles, what issues they would be comfortable discussing, what they considered to be the most important features of a social media peer-support site, and other feedback

^a^The other RCT (Gjerdingen et al., 2013 [67•]) is included as a mixed-methods trial in this table

^b^The other non-randomized trial (Lim et al., 2019 [66•]) is included as a mixed-methods trial in this table

^c^The other single-group studies (Campbell et al., 2019 [69•]; Dow et al., 2008 [68•]; Lim et al., 2020 [65•]) are included as mixed-methods trials in this table

**Table 2:** The study characteristics of single-group studies, stratified by study design

**Single-group study(s) (*n*** **= 4)**							
**Author, year, country**	**Participants**	**Setting**	**Aim**			**Follow up**	**Outcome measure(s)**
Wang, 2016, [70•] China	Individuals with experience(s) of traumatic event in the previous 3–60 months and reported 2 or more PTSD symptoms	- Urban sample: Internet advertisements - Rural sample: counselling centre in Sichuan province	(1) how urban and rural participants used the CMTR program, including general program usage and program adherence, (2) how program use was related to demographics (ie, sex, age, highest level of education attained, marital status, and annual family income), health problems (ie, PTSD and depressive symptom severity, trauma duration), psychological factors (ie, coping self-efficacy), and social factors (ie, social functioning impairment and social support after trauma) before the intervention, and (3) how program use was associated with change in outcomes after the treatment and at 3-months’ follow-up			Long-term follow up: 3-month	- Social isolation outcomes: Crisis Support Scale - Other outcomes: PTSD symptoms, depressive symptoms, trauma coping self-efficacy, social functioning and program use
Lee, 2018, [71•] Korea	Individuals (aged 20–65 years) diagnosed with PTSD following workplace accidents	Seoul National University Hospital and the Complex Regional Pain Syndrome Association in Korea	To investigate the effectiveness of an online imagery-based program for treating the psychiatric symptoms of patients with PTSD related to workplace accidents.			End of treatment: 4-week	- Social isolation outcomes: social support network via Functional Social Support Questionnaire (FSSQ) - Other outcomes: depressed mood, anxiety symptoms, PTSD symptoms, positive changes that occur after traumatic event, suicidal ideation
Goodwin, 2018, [72•] USA	Individuals (aged 18 years or older) with depressed mood or anhedonia and has visited primary care clinic in the previous 6 months	Primary care offices in urban and suburban areas in the USA	To examine helpfulness and safety of the Psycho-Babble			End of treatment: 6-week	- Social isolation outcomes: loneliness via loneliness item of Center for Epidemiologic Studies Depression Scale (CES-D-10), perceived social support via Perceived Social Support from Friends scale - Other outcomes: depression, self-efficacy, hopelessness, self-harm ideation, program ratings, rate of concerning content on the ISG
De Almeida, 2018, [73•] Portugal	Individuals (aged over 18 years) diagnosed with schizophrenia and attend psychosocial rehabilitation services	Outpatient mental health services centre in Portugal	To present the acceptability and model of analysis of weCOPE app			End of treatment: 8-week	- Social isolation outcomes: Social Support Satisfaction Scale - Other outcomes: recovery, empowerment, self-efficacy, personal and social performance, positive and negative symptoms
**Mixed-methods study(s) (*n*** **= 3)**							
**Author, year, country**	**Participants**	**Setting**	**Aim**	**Control group**	**Follow up**	**Data collection method**	
						**Quantitative: outcome measure(s)**	**Qualitative**
Loi, 2016, [74•] Australia	Older adults	Aged care specialized facility accommodating adults with a variety of psychiatric conditions	To investigate a structured training program on using the internet via touch technology (TT) to residents with psychiatric conditions living in residential care facility.	None	End of treatment: 6-week	- Social isolation outcomes: social isolation via Hawthorne Friendship scale - Other outcomes: self-esteem, familiarity of use and attitudes toward the Internet (via Internet questionnaire using 5-point Likert Scales)	Open-ended questions on the post-Internet questionnaire
Chen, 2020, [75•] Taiwan	Older adults (aged 65 years or above) with depressed mood	Long-term care facilities with more than 100 beds in Southern Taiwan	To investigate the effect of a social robot intervention on depression, loneliness, and quality of life of older adults in long-term care and to explore participants’ experiences and perceptions after the intervention.	None	- Mid-term follow-up: A week before 8-week observation (T1), end of 8-week observation (T2), mid-point of 8-week intervention (T3) - End of treatment: end of 8-week intervention (T4)	- Social isolation outcomes: loneliness via UCLA-LS-3 - Other outcomes: depressed mood, quality of life	Individual semi-structured interview to explore participants’ experience and perceptions of participating in the Paro intervention
Aschbrenner, 2016, [76•] USA	Adults (aged 21 or older) with a chart diagnosis of schizophrenia, schizoaffective disorder, MDD, or bipolar disorder; on stable pharmacological treatment and had body mass index (BMI) over 30	An urban community mental health center in southern New Hampshire, USA	To explore peer-to-peer support among individuals participating in a group lifestyle intervention that included social media to enhance in person weight management sessions.	None	End of treatment: 24-week	- Social isolation outcomes: social support from group members (Social Provisions Scale-Short Form SPS-10) - Other outcomes: perceptions of the group environment	- Qualitative focus group interviews to elicit participants’ perceptions of peer-to-peer support throughout the overall intervention

**Table 3:** The study characteristics of effectiveness trials, stratified by study design

**Fully powered randomized controlled trials (*n*** **= 3)**							
**Author, year, country**	**Participants**	**Setting**	**Intervention**	**Control/comparator condition**	**Follow up**	**Outcome measure(s)**	
Pot-Kolder 2018, [77•] Netherlands	Adults (aged 18–65) with a DSM-IV diagnosis of psychotic disorder, paranoid ideation in the past month and avoidance of shops, streets, public transport or restaurants	Dutch mental health centres	To establish the effectiveness of VR-CBT, compared with treatment as usual, in improving the quantity and quality of social participation in patients with psychotic disorders who experience paranoid ideation and social avoidance.	VR-CBT + treatment as usual (TAU) vs. waiting list + TAU only control group	- End of treatment: 3-month - Long-term follow up: 6-month	- Social isolation outcomes: amount of time spent with other people (assessed with Experience Sampling Method on a 7-point Likert Scale) - Other outcomes: momentary paranoia, perceived social threat, momentary anxiety, safety behaviour, paranoid thoughts, social interaction anxiety, social functioning, depression, quality of life and cognitive biases	
Moeini, 2019, [78•] Iran	- Female students (aged 15–18 years) with mild to moderate depression levels	Female high schools within Hamadan City, west of Iran.	To examine the effectiveness of a web-based intervention for depressive symptoms in female adolescents; Application of the SCT that to our knowledge is one of the first studies of this type.	Intervention vs. no-treatment control	- Mid-term follow up: 12-week - End of treatment: 24-week	- Social isolation outcomes: perceived social support via Farsi version of Perceived Social Support Scale-Revised (PSSS-R) - Other outcomes: depressive symptoms, self-efficacy, outcome expectations, self-regulation, website satisfaction and usage	
Kaplan, 2011, [79•] USA	Individuals diagnosed with schizophrenia spectrum or affective disorder	Mental health provider agencies, websites and e-newslists targeting individuals with mental illnesses	To examine the impact of unmoderated, unstructured Internet peer support on the well-being of individuals with psychiatric disabilities.	Peer support listserv vs. peer support bulletin board vs. waitlist control	- Mid-term follow up: 4-month - End of treatment: 12-month	- Social isolation outcomes: MOS-SSS - Other outcomes: recovery assessment quality of life, empowerment, depression and anxiety symptoms	
**Randomized comparative trials (*n*** **= 2)**							
**Author, year, country**	**Participants**	**Setting**	**Intervention**	**Control/comparator condition**	**Follow up**	**Outcome measure(s)**	
Van Voorhees, 2008, [80•] USA	Young people (aged 14–21 years) with persistent sub-threshold depression	Primary care practice sites in four USA states	To determine which primary care approach is more efficacious in reducing vulnerability of major depressive disorder as measured by pre/post changes in vulnerability factors.	CATCH-IT with brief advice from primary care physician (PCP-BA) vs. CATCH-IT with motivational interview (PCP-MI)	End of treatment: 4–8 weeks post-enrolment	- Social isolation outcomes: perceived family social support and perceived peer social support from the National Longitudinal Study of Adolescent Health (ADHEALTH) - Other outcomes: symptoms of other mental disorders with binary questions, depressive symptoms, closeness to parents, social acceptance, closeness to classmates and level of school impairment related to depressed mood	
Saulsberry, 2013, [81•] USA	Young people (aged 14–21 years) with persistent sub-threshold depression	Primary care sites across Southern and Midwestern USA	To determine one-year follow up outcomes of the CATCH-IT intervention	CATCH-IT PCP-MI vs. CATCH-IT PCP-BA	Long-term follow up: 1-year	- Social isolation outcomes: loneliness (single item rated on a 4-point scale) - Other outcomes: depressive symptoms clinically significant depressive episodes, self-harm ideation and hopelessness	
**Randomized controlled trials (not fully powered) (*n*** **= 3)**^a^							
**Author, year, country**	**Participants**	**Setting**	**Intervention**	**Control/comparator condition**	**Follow up**	**Outcome measure(s)**	
Marasinghe 2012, [82•] Sri Lanka	Individuals aged 15–74 years, having been admitted to the hospital after self-harm attempt and with significant suicidal intent	Colombo South Teaching Hospital, Sri Lanka.	To test whether a Brief Mobile Treatment (BMT) intervention can improve outcomes relative to usual care among suicide attempters	Immediate vs. delayed brief mobile treatment (IBMT vs. DBMT)	- Long-term follow up: 6- and 12-month	- Social isolation outcomes: Medical Outcomes Study-Social Support Scale (MOS-SSS) - Other outcomes: suicidal intent, depressive symptoms, alcohol use, drug use	
Kaplan, 2014, [83•] USA	Mothers over the age of 18 with a diagnosis of a mood or schizophrenia spectrum disorder	Websites and e-news lists of the Temple University Collaborative and partners	To explore the effect of an Internet educational intervention for mothers with serious mental illness.	Intervention vs active control	End of treatment: 3-month	- Social isolation outcomes: MOS-SSS - Other outcomes: parenting efficacy, parenting skills, coping skills, parental stress	
Interian, 2016, [84•] USA	- Veterans: veterans of Operation Iraqi Freedom (OIF)/Operation Enduring Freedom (OEF), with a positive screen on the PTSD Checklist (PCL) (≥50) - Family member/partner: designated by participants	VA New Jersey Health Care System	To assess a brief Internet-based intervention that provided Veterans’ families with psychoeducation on postdeployment readjustment	Control group VS intervention group	Long-term follow up: 2-month	- Social isolation outcomes (Veterans): perceived family support via Multidimensional Scale of Perceived Social Support (MSPSS) - Other outcomes (Veterans): mental health service use, perceived criticism - Other outcomes (family members): perceived criticism, family empowerment, perceived efficacy to carry out tasks targeted by the intervention (assessed with a 7-item measure)	
**Mixed-methods trial(s) (*n*** **= 1)**							
**Author, year, country**	**Participants**	**Setting**	**Aim**	**Control group**	**Follow up**	**Data collection method**	
						**Quantitative:outcome measure(s)**	**Qualitative**
Ellis, 2011, [85•] Australia	Undergraduate students (aged 18–25 years) suffering from low-to-moderate levels of distress	Department of Psychology and Faculty of Health Sciences at The University of Sydney	To assess the efficacy of a brief online CBT intervention (MoodGYM) compared with an online support group (MoodGarden) in decreasing symptoms of depression and anxiety, and improving dysfunctional thoughts, social support, and CBT literacy in young adults.	Online CBT (MoodGym) vs. online peer support (MoodGarden) vs. no-treatment control	End of treatment: 3-week	- Social isolation outcomes: Online Social Support Scale (OSSS) - Other outcomes: depression, anxiety, stress, automatic negative thoughts	- Open-ended questions to evaluate the best and worst aspects of the intervention

^a^The other not fully powered RCT (Ellis et al., 2011 [85•]) is included as a mixed-methods trial in this table

**Table 4:** Characteristics of interventions, stratified by types of interventions

Author, year	Study design	Intervention	Duration
**Web-based programmes (*n*** **= 16)**			
Wang, 2016 [70•]	Single-group	- The Chinese version of My Trauma Recovery (CMTR): web-based self-help intervention program - 6 modules offering education and exercises for trauma recovery-related topics	1 month
Rotondi, 2005 [54•]	Feasibility (pilot RCT)	- Web-based psychoeducation programme (the Schizophrenia Guide software) that provided online group therapy with individual patients or with support persons and educational materials - Online therapy groups were with a) support persons only, b) PWS only, c) multifamily therapy group for both PWS and support persons	(duration of intervention not specified)
Rice, 2020 [59•]	Feasibility (single-group)	- Entourage –online social anxiety intervention based on the Moderated Online Social Therapy (MOST) model (positive psychology, mindfulness and strength-based theories) - Features: an interface for users to build social connections; therapy comics and modules; a problem-solving discussion board - The therapy content is individually tailored to each participant by clinical moderators who can suggest specific content based on individual users’ treatment needs and goals. - Participants continued their in-person therapy at the same time at their local headspace centre	12 weeks
Rice, 2018 [60•]	Feasibility (single-group)	- Rebound – an online social therapy intervention programme based on the MOST model - Integrates social networking and individually-tailored interactive psychosocial interventions; helped users to identify key personal strengths using interactive online card-sort task and encourage users to put their strengths into action	12 weeks
O’Mahen, 2014 [55•]	Feasibility (pilot RCT)	- Netmums – a guided internet behavioural activation (BA) treatment - Online programme supplemented by resources on the Netmum website, online peer support and weekly phone call support from mental health workers	12 sessions
Moeini, 2019 [78•]	RCT	- The DAD (Dorehye Amozeshie Dokhtaran) website: depression improvement program - Based on Social Cognition Theory constructs - 7 modules in multimedia format with online assistance from psychiatrists, daily mood assessments and supplementary resources	6 months
Ludwig, 2020 [62•]	Feasibility (single-group)	- An online social media platform (‘Horyzons’) that integrates therapeutic content from CBT, positive psychology, mindfulness and meditation that can be used independently - To foster positive social connections among users, allowusers to discuss specific issues, receive support or suggestions and guided through problem-solving steps; track personal goals and share progress. - User content and activity suggestions are tailored to users’ individual strengths and goals	12 weeks
Lee, 2018 [71•]	Single-group	- Online imagery-based program - First phase: help patients to become more aware of their sensory experiences; second phase: mediate early-life trauma; third phase: address recent trauma; fourth phase: restore positive belief in oneself - Appropriate sound-enhanced imagery experiences aided relaxation and increased emotional impact of each treatment session	4 weeks
Kaplan, 2014 [83•]	RCT	- Internet-based parenting intervention - Experimental condition: online parenting course based in CBT techniques; peer support listserv via email - Active control condition: access to website with educational factsheets - Both groups continued to receive their usual health-care services	3 months
Kaplan, 2011 [79•]	RCT	- Listserv: unmoderated, unstructured Internet peer support Listserv (anonymous communication via group e-mail) - Bulletin board: unmoderated peer support	12 months
Interian, 2016 [84•]	RCT	- ‘Family of Heroes’ (FoH): Brief internet intervention - The training uses avatar characters that deliver psychoeducation and engage in simulated conversations concerning post-deployment stress and mental health treatment. - Stimulated conversations help family members choose statements that convey empathy and soften tone of conversation. Each conversational scenario focused on: de-escalating an argument, renegotiating household responsibilities, and encouraging VA mental health treatment-seeking.	1 h
Goodwin, 2018 [72•]	Single-group	- Psycho-Babble website: Internet Support Group (ISG) for depression - Provide fact-based information on mental health and access to a well-established ISG for primary care patients - Content based on National Institute of Mental Health (NIMH) and MoodGYM online intervention	6 weeks
Ellis, 2011 [85•]	Feasibility (pilot RCT)	- Online CBT (MoodGym): 5-module (over 3 sessions) self-help program to reduce dysfunctional thinking, overcome negative feelings and identify relaxation strategies - Online peer support (MoodGarden): online mental health resource offering peer-based support and information on self-management, participants can also share their experiences on a message board	3 weeks
Campbell, 2019 [69•]	Feasibility (single-group)	- 6 KHL Circles (groups) conducted over a 12-month period - For each Circle, KHL Counselors posted psychoeducational material about family discord weekly and encouraged discussion activity and interaction between participants to address issues within the topics.	8 weeks
Bailey, 2020 [63•]	Feasibility (single-group)	- Affinity: enhanced online social networking intervention - Follows the MOST model: peer social networking, problem-solving forum, therapeutic content delivered via comics	8 weeks
Alvarez-Jimenez, 2018 [64•]	Feasibility (single-group)	- MOMENTUM program: strengths and mindfulness-based intervention - Merges interactive psychosocial intervention modules and online social networking - Content suggestions for each user tailored weekly based on user’s needs, interests and strengths. - Participants continued treatment within PACE Clinic	2 months
**Phone-based interventions (*n*** **= 7)**			
Price, 2014 [61•]	Feasibility (single-group)	- Daily automated messages were sent to participants after their discharge from the hospital using the Connecting to Help After Trauma (CHAT) program - Themes of messages included re-experiencing, avoidance and hypervigilance to provide informational support and to assess trauma symptoms after their injury	15 days
Pfeiffer, 2017 [57•]	Feasibility (single-group)	- Automated phone call intervention - Participants received weekly visits or phone calls from family/friend or a peer support specialist - Patient monitoring and feedback facilitated by weekly automated phone calls. The phone system utilized interactive voice response technology (IVR) – based on patients’ responses to the assessments via the system, their support persons will guide their phone interactions with the patients - Continued usual outpatient mental healthcare after discharge	6 months
Lim, 2020 [65•]	Feasibility (single-group)	- + Connect app: users are to complete tasks which were delivered via: text and images, Shared Experience Videos featuring young people with lived experiences, Expert Videos featuring academics introducing core concepts, Actor Videos featuring actors modelling social behaviours. - The app is gamified to increase engagement. There is also a mood evaluation tracker	6 weeks
Lim, 2019 [66•]	Feasibility (single-group)	− + Connect app	6 weeks
Hanssen, 2020 [56•]	Feasibility (pilot RCT)	- Schizophrenia Mobile Assessment and RealTime feedback application (SMARTapp) - The app was personalised for all participants, according to their personal preferences (answered at baseline) so they could access their coping strategies, comforting thoughts and relaxing activities at any time in-app - All participants completed up to six short Experience Sampling Method (ESM) questionnaires daily.	3 weeks
Gjerdingen, 2013 [67•]	Feasibility (pilot RCT)	- Peer telephone support: peer supporters provided educational, emotional and comparison support - Postpartum doula group: face-to-face postpartum doula services (24 h of services over 6 weeks), including education regarding infant care, practical support and emotional support - It was expected that all 3 groups would receive usual depression treatment from their health care providers	3 months
De Almeida, 2018 [73•]	Single-group	- weCOPE mobile application - 4-module intervention – symptom monitoring, problem-solving, anxiety-management and goal setting	8 weeks
**Blended interventions (*n*** **= 7)**			
Van Voorhees, 2008 [80•]	RCT	- CATCH-IT (Competent Adulthood Transition with Cognitive-Behavioral and Interpersonal Training) programme: based on Behavioural Activation, Cognitive Behavioural Therapy (CBT), Interpersonal Psychotherapy (IPT) techniques and a community resiliency concept - Teaches adolescents how to reduce behaviours that increase vulnerability for depressive disorders and increase behaviours that are thought to protect against depression	14 modules
Van Voorhees, 2005 [58•]	Feasibility (single-group)	- Online programme based on Cognitive Behavioural Therapy (CBT), Interpersonal Therapy (IPT) - Initial motivational interview (MI) and follow-up MI with a primary care physician (PCP)	11 modules
Saulsberry, 2013 [81•]	RCT	CATCH-IT internet-based program	14 modules
Marasinghe, 2012 [82•]	RCT	- Mobile follow-up treatment of 12 months - Face-to-face component: mediation and interventions to increase social support, reduce alcohol use - Online components: phone calls at various follow-up time points post-discharge to assess suicidality and mood, plan intervention, provide guidance for social support; access to audio messages; weekly motivational messages up to 26 weeks - The BMT was administered in addition to usual care throughout the study	12 months
Loi, 2016 [74•]	Single-group	- Internet program based on a local program for older adults (Internet for Seniors) - Training program to teach older adults Internet-using skills, sending or receiving emails. Apple iPads were used as the Touchscreen Technology	6 weeks
Dow, 2008 [68•]	Feasibility (single-group)	- Participants were trained for basic computer operation, Internet searching, sending/receiving emails, virus protection and avoiding dangers. - Participants were given a recycled personal computer to keep after the study - The intervention was carried out in person, in groups	4 weeks
Aschbrenner, 2016 [76•]	Single-group	- Group-based lifestyle intervention - Face-to-face component: weekly in-person weight management sessions, optional twice weekly group exercise sessions - Digital component (introduced in week 6): use of technology and social media (private Facebook group) to facilitate monitoring and peer support. Participants to post content related to healthy eating and exercise or described personal successes or challenges towards achieving lifestyle goals. Study staff regularly posted content related to topics covered in the group sessions, reminders to exercise, and tips for healthy eating	24 weeks
**Socially assistive robot intervention(s) (*n*** **= 1)**			
Chen, 2020 [75•]	Single-group	- 24-h Personal Assistive Robot (Paro) intervention: - Each participant given a Paro to keep for the intervention stage, they were free to choose when to interact with it, to take the Paro outside or put it aside. - Paro is a kind of animal companion robot and has the appearance of a baby harp seal. It is equipped with tactile sensors that monitor sound, light and touch. It can show human-like emotional reactions	8 weeks of observation (usual care) and 8 weeks of intervention
**Virtual reality intervention(s) (*n*** **= 1)**			
Pot-Kolder, 2018 [77•]	RCT	- Sessions of virtual-reality-based cognitive behavioural therapy (VR-CBT) - Participants move within four virtual social environments (street, bus, café and supermarket) which were individualised to match their paranoid fears of the patient. - Patients and therapists communicated during VR sessions to explore suspicious thoughts and drop safety behaviours during social situations.	16 sessions over 8–12 weeks

**Table 5:** Results of the feasibility studies, stratified by study design

Author, year	N (n allocated to intervention, control)	Mean age (years)	Gender (% female)	Intervention effect on social isolation outcomes
**Randomized trial(s) (*n*** **= 4)**				
Rotondi, 2005 [54•]	30 PWS (i = 16, c = 14), 21 support persons (i = 11, c = 10)	37.5 (PWS), 51.52 (support persons)	70% (PWS), 66.7% (support persons)	- Comparing baseline and 3-month follow up, PWS in the telehealth group showed a trend towards greater social support (F(1, 27) = 3.79, *p* = .062). For support persons, there were no significant differences for social support (F(1, 18) = 0.36).
O’Mahen, 2014 [55•]	83 (i = 41, c = 42)	Not specified	100%	- At 17-week follow up, there were no between-group differences in perceived support scores (95% CI: − 1.79 to 2.03, *p* = 0.27), effect size is medium (d = 0.50, 95% CI 1.02 to − 0.02).
Hanssen, 2020 [56•]	- 64 - Final data analysis: 50 (i = 27, c = 23)	37.9 (i), 40.3 (c)	33.3% (i), 39.1% (c)	- There was no group-by-time interaction and no group effect on loneliness - Statistically significant decrease in loneliness scores over time in both groups (b = − 0.004, 95%CI: − 0.007 to − 0.0009, *p* = 0.01, d = − 0.11).
Gjerdingen, 2013 [67•]	39 (doula group = 12, telephone support group = 13, c = 14)	29.7 overall	100%	- No significant group differences in social support comparing baseline to 6-month follow up
**Non-randomized trial(s) (*n*** **= 2)**				
Pfeiffer, 2017 [57•]	48 (family/friend group = 19, specialist group = 29)	50.1 overall	25% overall	- No statistically significant differences between time points were found on perceived social support. - There were also no statistically significant differences between groups at 3 or 6 months for perceived social support
Lim, 2019 [66•]	20 (young people with SAD = 9, students with no mental health conditions = 11)	21 (SAD), 20.36 (students)	44.44% (SAD), 45.45% (students)	- SAD: loneliness scores decreased in a linear trend from baseline to 3-month follow up - Student: loneliness scores decreased from baseline to post-intervention and 3-month follow up - Across the entire group, loneliness showed mean negative slope (M = − 3.82, 95% CI: − 5.54 to − 2.17). On average, participants’ loneliness scores decreased by 7.64 points by follow-up (d = 0.94).
**Single-group study(s) (*n*** **= 10)**				
Van Voorhees, 2005 [58•]	14	Age range: 18–24 years overall	42.9% overall	- At post-intervention, there was a trend of increasing social support scores (d = 0.27, CI: − 0.73, 1.24, *p* = 0.13)
Rice, 2020 [59•]	89	19.8 overall	47.2% overall	- Comparing baseline and post-intervention, statistically significant improvements were found on social connectedness (d = 0.63, *p* < .001), significant decreases in loneliness (d = 0.63, p < .001). Changes in social network were not significant.
Rice, 2018 [60•]	42	18.5 overall	50% overall	- Comparing baseline and post-intervention, there was no significant increase in social connectedness (*p* = 0.711) or social support (*p* = 0.470)
Price, 2014, [61•] USA	31	37.1 overall	45.2% overall	- There was no reported analysis on differences of social support, but from the descriptive statistics table there is around a 2-point decrease on social support comparing baseline to 1-month follow up and baseline to 3-month follow up
Ludwig, 2020 [62•]	24	25.16 overall	36.8% overall	- Loneliness scores showed moderate reductions from baseline to mid-treatment (6-week) (d = 0.27). - Changes in participants’ perceived social support increased from baseline to 6-week and post-treatment (12-week) (d = 0.03 and d = 0.10), although modest and not maintained at 16 weeks.
Lim, 2020 [65•]	12	20.5 overall	25% overall	- The mean of slopes indicated loneliness scores were more likely to reduce after intervention (M = − 0.34, SD = 0.24). - Participants could be expected to have scores that are about 0.3 standard deviations lower at post-treatment, and about 0.6 standard deviations lower at 3-month follow-up than at baseline
Dow, 2008 [68•]	14	65.5 overall	86% overall	- There was a decrease of loneliness scores for 11 participants
Campbell, 2019 [69•]	105	16.2 overall	81.9% overall	- Due to the drop-off in response rates between the baseline survey (105/105, 100%) and final survey (8/105, 7.6%), data quality was too low to conduct meaningful analysis.
Bailey, 2020 [63•]	20	21.7 overall	55% overall	- Comparing baseline and post-intervention, differences on social connectedness were not statistically significant
Alvarez-Jimenez, 2018 [64•]	14	20.3 overall	78% overall	- Statistically significant improvements were found in subscales of social support at post-intervention: attachment (d = 0.70, *p* = 0.05) and guidance (d = 0.75, *p* = 0.03). - 33% of participants had a reliable decline on loneliness

**Table 6:** Results of the single-group studies

Author, year	N (n allocated to intervention, control)	Mean age (years)	Gender (% female)	Intervention effect on social isolation outcomes
**Single-group study(s) (*n*** **= 7)**				
Wang, 2016 [70•]	146 (urban = 56, rural = 90)	Age range: 16–70 overall	67.86% (urban), 82.22% (rural)	- At post-intervention, the use of the relaxation module was associated with negative change in social support (b = − 0.10, *p* = 0.04). Use of the triggers, self-talk, unhelpful coping and mastery tools modules were not associated with significant changes in social support - Total number of days using the program was positively correlated with social support scores (r = 0.22, *p* < 0.01)
Loi, 2016 [74•]	5	69.9 overall	40% overall	- There were no significant differences before and after the intervention for social isolation (t = − 2.434, *p* = 0.072)
Lee, 2018 [71•]	35	48.1 overall	14.3% overall	- Patients did not show significant improvements on FSSQ (t = 0.197, *p* = 0.84) at post-treatment
Goodwin, 2018 [72•]	34	32.53 overall	79.41% overall	- No significant changes were found in loneliness (*p* = 0.51) or social support (*p* = 0.91) at post-treatment
De Almeida, 2018 [73•]	9	38.11 overall	22% overall	- Statistically significant improvement at post-treatment was found in social support (*p* = 0.021). Improvements were also found in subscales (intimacy: *p* = 0.012; satisfaction with family: *p* = 0.026).
Chen, 2020 [75•]	20	81.1 overall	65% overall	- Comparing T2 to T4, statistically significant decreases in loneliness scores were found over time (F(3, 57) = 61.7, *p* < 0.001). - There were significant differences in every time point comparison: T2 vs T3 t = 8.84, p < 0.001, d = 1.95; T2 vsT4 t = 8.47, p < 0.001, d = 2.50; T3 vs T4 t = 2.48, *p* = 0.023, d = 0.75.
Aschbrenner, 2016 [76•]	25	48.6 overall	56% overall	- The global score for the assessing perceived social support from the group was high (M = 30.8 SD = 5.5).

**Table 7:** Results of the effectiveness trials, stratified by study design

Author, year	N (n allocated to intervention, control)	Mean age (years)	Gender (% female)	Intervention effect on social isolation outcomes
**Fully powered randomized controlled trial(s) (*n*** **= 3)**				
Pot-Kolder, 2018 [77•]	116 (i = 58, c = 58)	36.5 (i), 39.5 (c)	31% (i), 28% (c)	- VR-CBT did not significantly increase the amount of time spent with other people at post-treatment compared to baseline (d = 0.25; 95% CI: − 0.064 to 0.344, *p* = 0.178), but it did at 6-mont follow-up compared with baseline (d = 0.50, 95% CI: 0.072 to 0.502, *p* = 0.009). - Time spent with others decreased by 2.4% in the control group between baseline and follow up, whereas it marginally increased by 0.3% in VR-CBT
Moeini, 2019 [78•]	128 (i = 64, c = 64)	16.2 (i), 16.5(c)	100%	- In the intervention group, there was a statistically significant increase in social support from baseline to 24-week (*p* = 0.002). - Compared to the control group, improvements in social support from baseline to 12 weeks were found to be statistically significant (*p* = 0.011).
Kaplan, 2011 [79•]	300 (i = 200, c = 100)	47 overall	66.5% (i), 64% (c)	- There were no differences between conditions on social support scores (F(1,298) = 0.08; *p* = 0.93). - Across the entire sample, there was a statistically significant improvement on social support scores (F(1,298) = 3.16; p = 0.04)
**Randomized comparative trial(s) (*n*** **= 2)**				
Van Voorhees, 2008 [80•]	83 (motivational group = 43, brief advice group = 40)	17.44 overall	56.18% overall	- For perceived social support by peers, significant pre-post differences found across entire sample (p < 0.001), but no differences between groups (*p* = 0.62). Individually, both groups demonstrated increases with moderate to large effect sizes (MI: 0.76, 95% CI: 0.18, 1.32 p < 0.001; BA: 1.39, 95% CI: 0.75, 1.99, p < 0.001; all: 1.09, 95% CI: 0.64, 1.52) - There was no change in the perceived family support scale
Saulsberry, 2013 [81•]	83 (MI = 43, BA = 40); 58 overall at one-year follow up	17.26 overall	57% overall	- Significant decline in the mean rating of loneliness for the entire sample from baseline to one-year follow-up (ES = 0.49, 95% CI: 0.18, 0.79, p < 0.001). Individually, in both groups there were significant declines in the mean loneliness score from baseline to one-year follow-up (MI: ES = 0.43, 95% CI: 0.00, 0.85, p = 0.01; BA: ES = 0.54, 95% CI: 0.08, 0.98, p = 0.01).
**Randomized controlled trial(s) (not fully powered) (*n*** **= 4)**				
Marasinghe, 2012 [82•]	68 (i = 34, c = 34)	30 (i-male), 34(i-female), 29 (c-male), 31 (c-female)	50%	- Scores on social support improved significantly across time in both conditions - The BMT group also showed a significant improvement of social support when compared to the control group
Kaplan, 2014 [83•]	60 (i = 31, c = 29)	37 (i), 36 (c)	100%	- No significant differences were found between intervention and control groups on social support (t = 0.05, *p* = 0.96, d = 0.02)
Interian, 2016 [84•]	103 dyads formed of veterans and family members (i = 50, c = 53)	- Veterans: < 29 years (32.3%), 30–36 years (36.5%), 37 or older (31.3%) - Family members: < 29 years (35.2%), 30–36 (24.2%), 37 or older (40.7%)	- Veterans: 18.8% - Family members: 85.7%	- Across time, participants in the intervention group were significantly more likely to report a decrease in perceived family support (p = 0.04) compared to the control group
Ellis, 2011 [85•]	39 (MoodGym = 13, MoodGarde *n* = 13, c = 13)	19.67 overall	77% overall	- Online social support significantly improved for the MoodGarden (online peer support) group compared with both MoodGym (online CBT) and control (MoodGarden vs.control: t = 2.31, *p* = .03; MoodGarden vs. MoodGym: t = 3.62, *p* = .00).

## Results

**Fig. 1:**
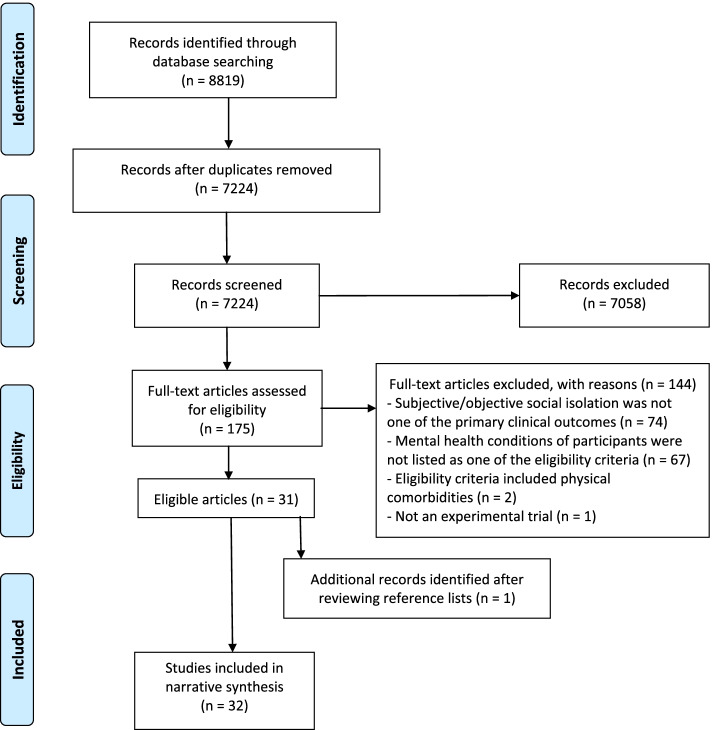
PRISMA flow diagram describing the selection of articles

We identified 8819 articles from the search of five databases. We conducted full text screening of 175 articles, finding 31 to be eligible. One other article was identified through reviewing the reference lists, bringing the total to 32 included articles (Fig. 1).

A total of 1945 participants were involved in the 32 studies, with individual sample sizes ranging from 4 to 300.^1^ Twenty-six studies had fewer than 100 participants. The target populations involved individuals with depressive symptoms (*n* = 12) [55•, 57•, 58•, 60•, 63•, 67•, 68•, 72•, 75•, 78•, 80•, 81•], psychotic symptoms (*n* = 7) [54•, 56•, 62•, 64•, 66•, 73•, 77•], trauma (*n* = 4) [61•, 70•, 71•, 84•], social anxiety disorder (*n* = 2) [59•, 65•], suicidal intent (*n* = 1) [82•] and elevated levels of distress (*n* = 1) [85•]. Five other studies involved participants with a variety of mental health conditions, including bipolar disorder, mood disorders and affective disorders [69•, 74•, 76•, 79•]. Some studies recruited individuals from more than one diagnostic group, for example individuals with diagnosis of schizophrenia, major depressive disorder, or bipolar disorder [76•], or individuals with schizophrenia or affective disorder [79•].

Of the total of 32 studies, 16 (50%) were feasibility or pilot studies [54•, 55•, 56•, 57•, 58•, 59•, 60•, 61•, 62•, 63•, 64•, 65•, 66•, 67•, 68•, 69•]. Most were uncontrolled, pre-post measures pilot studies and four were pilot RCTs [54•, 55•, 67•, 73•]. We grouped these studies as feasibility studies as their primary aim was to assess the feasibility and preliminary evidence of conducting future definitive trials utilizing the same interventions [86]. All feasibility studies also reported effectiveness outcomes. Seven other studies (21.9%) [69•, 70•, 71•, 72•, 73•, 74•, 75•] used the single-group/pre-post measures design. Nine included studies were effectiveness trials (28.1%) [77•, 78•, 79•, 80•, 81•, 82•, 83•, 84•, 85•], of which three were fully powered RCTs and six were RCTs that were not fully powered; all with the primary aim of assessing intervention effectiveness. Overall, nine of 32 studies used mixed methods (36%).

Twelve studies were carried out in the United States [54•, 57•, 58•, 61•, 67•, 72•, 76•, 79•, 80•, 81•, 83•, 84•], 11 in Australia [59•, 60•, 62•, 63•, 64•, 65•, 66•, 68•, 69•, 74•, 85•], two in Netherlands [54•, 56•], one in China [70•], one in Iran [78•], one in Korea [71•], one in Portugal [73•], one in Sri Lanka [82•], one in Taiwan [75•] and one in the United Kingdom [55•]. Only four studies (12.5%) were conducted between 2000 and 2010 [54•, 58•, 68•, 80•]; the others were conducted between years 2011–2020, among which 21 studies were conducted within the last 5 years (2016–2020) at the time of conducting this review.

The interventions evaluated used technology to relay therapeutic content, to provide social support and as a tool for distance follow-up or monitoring. Although there were interventions designed specifically to reduce loneliness (*n* = 2) [65•, 66•], most aimed to promote the well-being of participants, such as improving social functioning or mental and physical health outcomes. In these studies, social isolation was not the primary outcome, but was included among a range of psychosocial outcomes instead. Only four studies explicitly stated their primary and secondary outcomes – among these, one listed loneliness and perceived social support among their primary outcomes [62•], and another listed objective social participation [77•]. Other studies did not explicitly differentiate between primary and secondary outcomes, although six studies did lay out their primary objectives/hypotheses [63•, 65•, 66•, 67•, 79•, 83•].

Among the digital elements incorporated, most studies reported using asynchronous digital content (communication between participants and clinicians not occurring at the same time), including online intervention programs [54•, 55•, 58•, 70•, 78•, 79•, 80•, 81•, 83•, 84•, 85•], peer support via email listservs [79•] and symptom monitoring via text messages or smartphone apps [56•, 61•, 62•, 66•, 73•, 82•]. Some studies reported enhancing peer support via online chatroom features and social networking elements in their interventions [59•, 60•, 62•, 63•, 64•], but it is unclear whether the use of these online social support elements was synchronous (live communication happening in real time). Only three studies stated clearly that the intervention incorporated a synchronous digital component, in which participants received real time peer support via phone calls [57•, 67•] or received therapeutic feedback in virtual reality sessions with a clinician [77•].

Of the 32 studies, 29 assessed subjective social isolation, two measured objective social isolation [71•, 77•] and one assessed both subjective and objective social isolation outcomes [59•]. Overall, loneliness and perceived social support were the most assessed dimensions of social isolation, whereas for objective social isolation specifically, social network size and time spent with others were assessed. Commonly used validated outcome measures included the: University of California Los Angeles Loneliness Scale (UCLA-LS) (*n* = 7) [59•, 62•, 64•, 65•, 66•, 68•, 75•], Medical Outcomes Trial-Social Support Scale (MOS-SSS) (*n* = 7) [54•, 60•, 61•, 67•, 79•, 82•, 83•], Social Provisions Scale (SPS) (*n* = 4) [55•, 62•, 64•, 79•], Multidimensional Scale of Perceived Social Support (MSPSS) (*n* = 3) [57•, 69•, 84•] and Social Connectedness Scale (SCS) (*n* = 2) [59•, 63•]. Seven studies used more than one social isolation measure [54•, 59•, 60•, 62•, 64•, 67•, 72•]. Four studies used unvalidated outcome measures including single-item measures, multiple-item measures and questionnaires administered via the Experience Sampling Method (ESM) [54•, 56•, 67•, 81•]. Detailed characteristics of studies, including study aims, sample sizes, study designs and outcome measures, are outlined in Table 1 (feasibility studies), Table 2 (single-group studies) and Table 3 (effectiveness trials).

To answer our research questions, we structured our findings based on 1) nature of the interventions evaluated, 2) quality assessment, and 3) strength of evidence. We first describe the range of intervention types and their main components, then summarise the available evidence on feasibility and on effectiveness.

### Types of intervention

Characteristics of all interventions are outlined in Table 4, stratified by types of intervention.

#### Web-based interventions

In this review, we adopted the definition of a web-based interventions as ‘a primarily self-guided intervention programme that is executed by means of a prescriptive online programme operated through a website and used by consumers seeking health- and mental-health related assistance’ [87]. Overall, 16 out of 32 studies (50%) utilized web-based programmes, including eight feasibility/pilot studies [54•, 55•, 59•, 60•, 62•, 63•, 64•, 69•], three pre-post measure studies [70•, 71•, 72•], and five effectiveness trials [78•, 79•, 83•, 84•, 85•]. Of the eight feasibility studies, two were pilot RCTs that mainly aimed to examine feasibility and acceptability outcomes [54•, 55•]. All studies delivered web-based programmes in a multicomponent format.

Access to therapeutic content, peer support and clinical/peer moderation were frequently reported as part of the intervention. Therapeutic content was delivered in forms of therapy courses or modules consisting of core concept explanations, skill- or strength-building exercises and psychoeducational resources. Some also incorporated interactive content in forms of online games or comic strips [59•, 60•, 62•, 63•, 64•]. All studies provided therapeutic content to various degrees, including studies that assessed internet peer support groups [72•, 79•, 85•]. As an example, Goodwin et al. (*n* = 34) [72•] mainly investigated the role of online peer support via an established Internet Support Group (ISG) called the ‘Psycho-Babble’. Participants, with depressive symptoms, were given access to an Internet portal that contained the ISG as well as fact-based content related to mental health and depression.

Peer support was also a common element, incorporated in forms of group discussion forums, online chatrooms, existing social media (e.g., Telegram®) and email listservs. Online peer support was the main element investigated in three studies [72•, 79•, 85•] whereas nine other studies also incorporated peer support as part of the interventions, but not as the main element [55•, 59•, 60•, 62•, 63•, 64•, 69•, 78•]. Participants were able to interact with peers who had experienced the same difficulties using the online platforms under safety monitoring from the researchers. This was proposed to promote a sense of community, thus fostering social support and mental wellbeing.

We also identified eight studies that explicitly reported embedding moderation by peers [55•, 59•, 60•, 62•, 63•, 64•, 69•, 78•]; six of these also incorporated clinical moderation by professionals [59•, 60•, 62•, 63•, 64•, 69•]. The researchers reported the role of peer moderators to be promoting engagement and providing support when needed. Expert moderators were tasked with providing clinical guidance, monitoring the clinical status of participants and monitoring the safety of the online environment. Most other studies also made mention of monitoring from experts or clinicians. Among the three studies that did not report clinical or peer moderation, one was an underpowered RCT by Kaplan and colleagues (*n* = 60) [83•] which aimed to examine unmoderated and unstructured internet support for participants with schizophrenia or affective disorders. In the other two studies, interventions were designed as self-help, take-home resources [70•, 71•], thus the research focus was on assessing treatment courses that could be entirely self-facilitated by the participants. There was no mention of expert moderation/monitoring or additional social support component in either of these studies. For example, Lee et al.’s study (*n* = 35) [71•], conducted in South Korea, consisted of an online imagery-based program that was developed based on the cognitive model of PTSD. The main therapeutic objective was to utilize guided imagery techniques to help participants modify traumatic memories. Training sessions were made up of auditory guidance and background music to enhance imagery experience, supplemented by mind-body training techniques such as relaxation and meditation.

Apart from two studies [54•, 69•], the online programmes were individual-level interventions and were self-directed by the participants. Campbell et al.’s study (*n* = 105) [69•], the only feasibility study that recruited more than 100 participants, used an online platform (‘Kids Helpline Circle’) to conduct group counselling with young adults with mild depressive or anxiety symptoms. Rotondi et al. (*n* = 30) [54•] conducted therapy groups facilitated by mental health professionals using online bulletin boards. This feasibility study involved persons with schizophrenia and provided problem-solving group therapy to the participants and their family members. In both studies, while the online counselling/therapy was facilitated by the researchers, participants had access to other aspects of the intervention that also contained psychoeducational resources.

In 10 of the 16 studies (62.5%), researchers aimed to develop their interventions based on specific theoretical frameworks, including Cognitive Behavioural Therapy (CBT) [83•, 85•], Social Cognitive Theory (SCT) [78•], Behavioural Activation (BA) [55•] and the cognitive model of PTSD [71•]. It is also noteworthy that the web-based programmes (‘MOMENTUM’, ‘Affinity’, ‘Horyzons’, ‘Rebound’ and ‘Entourage’) in five feasibility studies [59•, 60•, 62•, 63•, 64•] were based on the MOST (Moderated Online Social Therapy) model developed by Orygen, a mental health organization based in Australia. The MOST model has its theoretical basis in Self-Determination Theory, positive psychology and mindfulness [88]. The interventions incorporated self-help therapy modules, a moderated social network, and personalised suggestions for therapy content from clinicians. Behavioural prompts were also included as a feature of persuasive systems – recommendations for behaviour change were given to users to implement in the real world. These interactive online platforms aimed to foster self-efficacy and social support for users in a safe environment. They are intended to supplement face-to-face treatments, therefore participants in these studies are recruited from local clinics and continued their in-person treatments during the study periods.

#### Telephone-based programmes

We identified seven studies, including six feasibility studies [56•, 57•, 61•, 65•, 66•, 67•] and one single-group study [73•] that delivered interventions using mobile telephones. Of the six feasibility studies, two were pilot RCTs with the primary aim to test feasibility and acceptability outcomes [56•, 67•]. More specifically, four utilized smartphone apps [56•, 65•, 66•, 73•], two used telephone calls [57•, 67•], and the other used text messages [61•]. Studies that utilized smartphone apps mentioned monitoring by clinical professionals while the others did not. Although each intervention provided certain degrees of therapeutic support that had their basis in concepts such as positive psychology, the theoretical basis for the formation of these interventions was not outlined in detail.

Of these studies, six studies shared a common component of symptom assessment and monitoring [56•, 57•, 61•, 65•, 66•, 73•]. Symptom monitoring was a feature in Hanssen and colleagues’ (*n* = 64) [56•] pilot feasibility RCT conducted with individuals with schizophrenia in the Netherlands. The researchers assessed the SMARTapp (Schizophrenia Mobile Assessment and RealTime feedback application), which was mainly designed for participants to carry out real-time monitoring of their symptoms by answering multiple questionnaires daily using the app. The app was personalized for the users at baseline assessment – e.g., users had in-app access to their preferred relaxation activities or comforting thoughts at any time. Participants in the intervention group received additional personalized feedback (suggestions for a certain activity or behaviour change) from the SMARTapp based on their questionnaire responses, whereas those in the control group used the app without individual feedback. In another study, Price et al. (*n* = 29) [61•] assessed a symptom-monitoring intervention utilizing text messages. Recruited after experiencing a traumatic injury, participants received daily messages that contained assessments of different symptom domains, including social support, hypervigilance and re-experiencing. Symptom assessment and monitoring was the main objective for these studies. The main aim of the +Connect app, which was used in two other feasibility studies [65•, 66•], was to reduce loneliness by conveying evidence-based concepts of positive psychology to users, in order to build on their strengths and empower them to engage in meaningful social interactions. Content was delivered in a multimedia format (via short videos or via text and images) and participants had to answer content-based quizzes afterwards. Through answering questions, completing daily mood tracking and progressing through various levels of content, participants also obtained points and badges. The addition of this gamification element was intended to promote participants’ engagement.

Social support was integrated in the two studies utilizing phone calls [57•, 67•]. In a pilot feasibility RCT, Gjerdingen et al. (*n* = 39) [67•] compared the effects of support from peer volunteers via phone calls versus in-person support from certified postpartum doulas versus usual care. In contrast, Pfeiffer et al. (*n* = 48) [57•] assessed a more complex digital intervention: the researchers used an automated telephone monitoring system to assess veteran patients’ depressive symptoms and medication adherence, while also providing social support to the participants via weekly phone calls/meetings with either a friend, a family member or a trained and certified peer specialist, depending on the participant’s choice. The automated phone system utilized interactive voice response technology (IVR) and consisted of scripted voice recordings to which participants responded. The family member/friend received a report on the participant’s responses and tips on how to interact with the participant during the meetings, while the peer specialist was instructed to guide interactions based on the participants’ responses using their own skills.

#### Blended interventions

A total of seven studies employed a blended approach by combining digital with face-to-face components in therapy. These were two feasibility studies [58•, 68•], two single-group studies [74•, 76•] and three effectiveness trials [80•, 81•, 82•]. The role of the digital component varied – some studies utilized technology as the main instrument to convey therapeutic content (*n* = 3) [58•, 80•, 81•] while in others it served as a monitoring/follow-up tool (*n* = 2) [76•, 82•]. Two other studies [68•, 74•] conducted in-person, group training sessions during which participants were trained to use a computer and the Internet. As an example, Dow and colleagues (*n* = 14) [68•] conducted a computer-training programme for rural carers with subsyndromal depressive symptoms. Participants were given a recycled personal computer and trained on how to use the computer, send and receive emails and to navigate the Internet. It was proposed that such training programmes may facilitate more social connections for the participants as they learn to connect with others online, thus reducing social isolation.

Another three studies – a feasibility study [58•] and two randomized comparative trials (not fully powered) [80•, 81•] – examined the effects of using face-to-face motivational interviews (MI) to complement the web-based programme (Competent Adulthood Transition with Cognitive-behavioural and Interpersonal Training – CATCH-IT), which was based on CBT and Interpersonal Therapy (IPT) principles. The web-based programme was the main component, while an in-person, 10- to 15-min MI was conducted once before and once after the online intervention. CATCH-IT was used for depression prevention with adolescents who had been experiencing persistent sub-threshold depressive symptoms, whereas the role of MI was proposed to enhance the users’ willingness for behaviour change, leading to increased effectiveness of the internet intervention.

It was unclear from the description of most studies whether there was any moderation for the digital component. In contrast, Aschbrenner and colleagues (*n* = 25) [76•] reported safety monitoring of the private Facebook group that participants were introduced to in their lifestyle intervention. This was a group intervention designed to promote the overall well-being of participants via weekly weight management sessions and the use of social media. Along with Marasinghe et al.,‘s (*n* = 68) [82•] RCT, the digital components in this study were implemented during more than half of the intervention period and served as an adjunct to complement the face-to-face intervention. The main purpose of the technology components was to monitor or facilitate participants’ self-monitoring of symptoms, as well as provide informational and social support.

Although there was some level of description regarding the theoretical basis of each individual component, the theoretical basis of using a blended approach of these components was unclear.

#### Social robots

Socially assistive robots are designed to interact with people in a socio-emotional way during interpersonal interactions to improve recovery and health outcomes [89]. Recently social robots have been increasingly utilized to alleviate psychological distress and reduce social isolation among elderly adults with dementia [90]. In this review, we identified one single-group study conducted by Chen et al. (*n* = 20) [75•] who delivered a social robot intervention involving elderly adults with depression. Participants were each allocated an animal companion robot (Personal Assistive RobOt – ‘Paro’) to keep throughout the intervention. They were encouraged to interact with it through touch and verbally, whenever they preferred to. Paro is shaped like a baby harp seal, equipped with tactile sensors and could show human-like emotional reactions. Paro and other animal robot interventions are aimed at encouraging human-animal interactions that can improve psychological and social functioning through the comfort and emotional attachment derived from close interactions and commitment to the animal companion robot.

#### Virtual reality

Virtual reality (VR) is utilized in therapy by allowing participants to complete therapeutic exercises in the virtual social world with the guidance of a therapist. In a fully powered RCT, Pot-Kolder and colleagues (*n* = 116) [77•] assessed the effectiveness of a virtual-reality-based CBT (VR-CBT) to enhance positive social participation (operationalised as time spent with others) among individuals with psychotic disorders who experienced paranoid ideation and social avoidance. Participants communicated with therapists in each session where they were exposed to virtual simulations of social situations. Based on individualised case formulations, each participant experienced exposure to different social environmental cues that elicited paranoid thoughts and safety behaviours. The participants worked with the therapists during these sessions to explore and challenge their safety behaviours and negative thoughts.

### Quality assessment

Out of the 16 included feasibility studies (all of which provided data collected with aim of assessing effectiveness) we judged six to be at moderate risk of bias, five at low risk of bias and five at high risk of bias. None of these studies accounted for confounders in the study design or data analysis. Of these 16 studies, five did not report complete outcome data (completion rates below 80%) and one did not specify completion rates.

Of the seven single-group, pre-post measures studies, four were judged to be at moderate risk of bias, two at low risk of bias and one at high risk of bias. Of these seven studies, two did not report complete outcome data and three did not specify completion rates.

There were three fully powered RCTs, one of each judged to be at high, moderate and low risk of bias respectively. The six other randomised trials that were not fully powered were all judged to be at moderate risk of bias. Eight out of nine randomized trials described methods of randomization. However, descriptions of concealment and blinding were unclear or missing on six randomized trials. Out of nine randomized trials, all specified completion rates but three did not report complete outcome data. Results of quality assessment are presented in Supplementary file 2.

### Strength of evidence

Although the heterogeneity of studies and lack of studies assessing social isolation as the primary outcome (in contrast, being included among a range of outcomes instead) make it difficult to form firm conclusions, we present a summary of the efficacy and feasibility outcomes of the included studies to provide a preliminary overview of the current state of evidence, categorised by respective study designs. We also outlined the participants’ characteristics, effectiveness and feasibility outcomes (for feasibility studies only) as well as intervention duration in Tables 5, 6 and 7 (for feasibility studies, single-group/pre-post measures studies and effectiveness trials respectively).

#### Feasibility and pilot studies

While their primary stated purpose was to obtain data to test the feasibility of conducting a full trial, all 16 feasibility studies aimed to provide data relevant to effectiveness. In a pilot RCT, Hanssen and colleagues (*n* = 64) [56•] found statistically significant decreases in loneliness scores across all participants using the SMARTapp intervention, but no difference was found between the intervention and control groups, hence effectiveness is not known. Rotondi and colleagues (*n* = 51) [54•] showed a non-significant trend towards greater perceived social support for participants in the web-based intervention group compared to the control group. The other two pilot RCTs [55•, 67•] did not report significant findings.

Although Van Voorhees and colleagues (*n* = 14) [58•] found a non-significant trend toward increasing social support scores among participants, this was a single- group study that did not include a control group to compare the effects of the online CATCH-IT intervention and so does not pertain to effectiveness. Using an online intervention based on the MOST model (‘Entourage’) and single-group design, Rice and colleagues (*n* = 89) [59•] showed statistically significant improvements in social connectedness and loneliness among participants at post-intervention compared to baseline, but no significant differences on social network scores; the lack of a control group means no conclusions can be drawn regarding effectiveness. Three studies did not report tests of significance but reported improvements in social isolation outcomes using different statistical tests [62•, 65•, 66•], such as latent trajectory models. The other seven single-group feasibility studies reporting effectiveness data did not find significant changes in social isolation outcomes [55•, 60•, 63•, 64•] or did not report statistical analysis testing for significant differences [61•, 68•, 69•].

Feasibility outcomes from the studies generally favoured proceeding to a full trial, as most studies (*n* = 11) [55•, 56•, 59•, 60•, 62•, 63•, 64•, 65•, 66•, 67•, 68•] reported satisfactory retention rates at above 70%. Among these, all studies utilizing web-based programmes based on the MOST model (*n* = 5) [59•, 60•, 62•, 63•, 64•] reported retention rates over 80%. Another pilot feasibility RCT conducted by Gjerdingen et al. (*n* = 39) [67•] compared the effects of telephone peer support vs face-to-face postpartum doula support and reported retention rates to be over 90%. However, the only feasibility study that recruited more than 100 participants reported a dropout rate as high as 92.4% [69•]. The study sought to assess an online group counselling platform (Kids Helpline Circle).

Acceptability outcomes were difficult to compare as different scales and methods of rating were utilized in each study. Generally, satisfaction ratings and feedback regarding the usefulness/helpfulness of the interventions were favourable, but there were also some mixed findings. For example, participants in Hanssen’s study (*n* = 64) [56•], with retention rate over 70%, rated the SMARTapp as easy to use (94%) and appealing (95%) but also annoying (38% in the comparison group and 73% in the intervention group) due to the frequent reminder beeps to prompt questionnaire completion. In two out of seven studies (28.6%) that involved both digital and face-to-face components, researchers reported lower satisfaction ratings for the digital component. This was observed in Van Voorhees and colleagues’ blended intervention (*n* = 14) [58•] and Gjerdingen et al’s (*n* = 39) [67•] study comparing telephone peer support vs face-to-face postpartum doula support. Lastly, eight feasibility studies also assessed safety outcomes [59•, 60•, 63•, 64•, 65•, 66•, 72•, 79•]. None reported adverse events that were attributed to the interventions assessed. All eight studies reported that all participants reported feeling safe while using the interventions.

Our reporting of findings from feasibility studies was intended to help identify new approaches for future research. Whilst findings demonstrate the variety of potentially feasible and acceptable interventions to be tested in large-scale trials, we cannot form firm conclusions about the interventions’ effectiveness in improving social connectedness and loneliness.

#### Uncontrolled studies providing pre/post comparisons

Mixed findings were demonstrated from the seven single-group studies. Chen and colleagues (*n* = 20) [75•] found significant decreases in loneliness scores among the elderly participants in the middle of, and at the end of, the 8-week social robot intervention compared to pre-intervention. De Almeida and colleagues (*n* = 9) [73•], using the weCOPE application, also demonstrated statistically significant increases in social support among individuals with schizophrenia over 8 weeks. Due to the lack of control groups in these studies, the findings should be taken, at best, as an indicator for a possibility of an intervention effect.

Among the other studies, one did not conduct a statistical test of significance [76•], while three did not find significant changes in the social isolation outcomes [71•, 72•, 74•]. Wang and colleagues [70•] reported correlations between usage and social support scores. Using a web-based intervention (the Chinese version of My Trauma Recovery - CMTR), the authors found statistically significant negative findings: decreases in social support scores correlated with increased uses of one of the program modules (the relaxation module), but only on the first day of the intervention, and there was no control group to compare this to. There were no significant correlations between social support scores and the use other five modules. Of all the studies, there was no report of adverse events related to the respective interventions. Together these studies do not provide strong evidence to support any of the interventions evaluated given that they lack control or comparison conditions.

#### Effectiveness trials

Three fully powered RCTs showed varying findings. Moeini et al. (*n* = 128) [78•] reported statistically significant improvements in social support among female adolescents with mild to moderate depressive symptoms in the intervention group compared to controls after a 24-week web-based intervention (Dorehye Amozeshie Dokhtaran – DAD). Conversely, studies from Pot-Kolder et al. (*n* = 116) [77•] and Kaplan et al. (*n* = 300) [79•] reported no significant differences between treatment and control groups. Pot-Kolder et al. [77•] conducted a VR-CBT intervention with participants with psychotic disorder, while Kaplan et al. [79•] involved individuals diagnosed with schizophrenia or an affective disorder to compare social support outcomes between three conditions: Internet peer support via a listserv, Internet peer support via a bulletin board, and a waitlist control condition.

Two randomized comparative trials that were not fully powered used blended interventions. Both trials did not find significant differences between the comparison groups. Involving adolescents at high risk of depression, Van Voorhees and colleagues (*n* = 83) [80•] compared CATCH-IT with motivational interviewing (MI) and CATCH-IT with brief advice. Similarly, Saulsberry et al. (*n* = 83) [81•] examined the effects of CATCH-IT with MI vs CATCH-IT with brief advice in a one-year follow up study of a RCT that also investigated the CATCH-IT intervention. As these studies did not report significant differences between intervention and control groups, we cannot reach conclusions about clinical effectiveness of the interventions.

The four pilot RCTs included also reported mixed findings. Kaplan et al. (*n* = 60) [83•] reported no significant differences in social isolation outcomes after an online parenting intervention involving mothers with mood disorder and schizophrenia. In a web-based intervention involving university students with elevated levels of distress, Ellis and colleagues (*n* = 39) [85•] found that perceived social support scores among participants in the online peer support group were significantly improved compared to online CBT and no-treatment control groups. Using a blended intervention involving individuals with suicidal ideation, Marasinghe et al. (*n* = 68) [82•] also reported significant improvements in social support in the intervention group compared to waitlist control. However, this study did not clarify their threshold of significance or *p*-value. Lastly, Interian and colleagues (*n* = 206) [84•] found that the veteran participants in the intervention group were significantly more likely to report a decrease in perceived family support compared to the control group. This was demonstrated at a 2-month follow up after a single-session one-hour web-based intervention (‘Family of Heroes’). Clear conclusions cannot be drawn due to the small sample sizes and lack of power calculations in the trials. Regarding safety, none of these four pilot RCTs reported adverse events related to the interventions in each study.

## Discussion

Given the rapidly evolving field of digital technology, this scoping review aimed to fill a gap in the evidence base by drawing together current findings on the effectiveness and feasibility of digital interventions for subjective and objective social isolation among individuals with mental health conditions. We found a very diverse body of literature: the characteristics of interventions and data analysis methods were found to vary across studies; participants were also from different age groups, recruited from a variety of settings and had a range of mental health conditions.

Among all included studies, half used web-based interventions while others used phone-based interventions (i.e., via smartphone applications, phone calls or text messages) or blended interventions combining digital and face-to-face components. The main components of the interventions included peer support, therapeutic content and symptom management. There were also two studies utilizing socially assistive robots and virtual reality respectively, demonstrating very limited evidence for the use of these technologies in this field. While VR is increasingly being used with individuals with mental health conditions, particularly those with a history of trauma [91], most studies did not investigate its effectiveness in relation to social isolation outcomes [92, 93]. Instead, the main focus was placed on other psychological outcomes such as anxiety and stress. Similarly, while previous research has consistently found positive effects from using socially assistive robots such as the robotic seal ‘Paro’ [94] and the robotic dog ‘Aibo’ [95] to tackle social isolation among the elderly, Chen and colleagues’ [75•] study, included in our review, was the first to use Paro in an intervention with individuals with mental health conditions.

The studies described in our scoping review identified a variety of approaches that have been successfully implemented in research and appear feasible. Hence, we propose that there is a range of different potential mechanisms for the further development and testing of digital interventions for social isolation in a mental health context. Regarding clinical effectiveness, we cannot draw conclusions for the overall effectiveness of digital interventions to alleviate social isolation from results of the included studies. Only one out of three fully powered RCTs [78•] delivering a web-based intervention provided significant supportive evidence that digital interventions may be able to reduce subjective social isolation. For the other studies, feasibility studies suffer from power limitations while many single-group studies and effectiveness trials were of low to moderate quality due to small sample sizes, lack of power calculations and lack of control groups. Evidence for alleviating objective social isolation is particularly insufficient due to the small number of studies that included it as an outcome, none of which reported significant findings. Nevertheless, the low proportion of high-quality studies in this review was balanced against the wide inclusion criteria to identify potentially promising approaches. Although not designed to evaluate clinical effectiveness and not generally demonstrating this, most included feasibility studies reported favourable retention rates and satisfaction ratings, particularly in those using smartphone apps and those utilizing web-based interventions based on the MOST model. Despite being at relatively higher risk of bias in study design features, the inclusion of these feasibility studies allowed us to identify feasible approaches to be tested in future research in an emerging field.

In the studies included in our review, there was a lack of evidence comparing different elements in each type of digital intervention, despite the promising variety of approaches. While synchronous communication has been well-described in the literature and is associated with good psychological outcomes [96], asynchronous communication in digital mental health care is not as sophisticated as that used in other clinical fields such as cardiology, radiology and dermatology [97]. Most included studies in this review used asynchronous technologies, yet none of the studies considered the differential effects that asynchronous and synchronous technologies might have had, including clinical effectiveness, adherence to the treatment or strength of therapeutic alliance between clients and clinicians [98, 99]. We also noted very little discussion within included studies about the differential effects of in-person versus digital elements in blended interventions. This aligns with the current state of the evidence, as little is known about the optimal ratio of face-to-face and digital elements in mental health interventions, or the compatibility of different in-person and digital components [100]. For future research, adequately powered trials could be carried out to distinguish these effects, to provide insight on how integrating different elements could impact the mechanisms and outcomes of each intervention.

We also identified a gap in the evidence regarding theory-based approaches, as half of the studies included did not specify a theory on which the interventions were based. None of the interventions using social robots, phone calls/messages and internet training programmes described theoretical bases. Among the 16 studies that conducted a theory-driven intervention, five were based on the self-determination theory (SDT) [59•, 60•, 62•, 63•, 64•], which proposes that developing a sense of autonomy, competence and relatedness are critical to the process of health behaviour change [101]. According to this model, treatment environments that enhance autonomy, competence and relatedness lead to increased engagement in the intervention, and in turn, result in better health outcomes and social functioning [88]. The shared components of these interventions include individually tailored therapy content, online social network and safety moderation. The clinical moderators of these studies also checked-in regularly with inactive participants to encourage their involvement with the intervention. The act of regular check-ins, as well as implementation of tailored content by moderators or clinicians, is also an element of persuasive technology, which previous research has also found to be effective in increasing user engagement [102, 103]. In all five trials, the retention rates were promising (over 80%), demonstrating support for the theory to facilitate engagement with the interventions. However, their clinical effectiveness of the interventions remains uncertain, as these studies did not include control or comparator groups. A few trials described approaches such as CBT and mindfulness theories underlying the design, whereas others were vague in articulating a theoretical basis. This lack of theory-driven approaches is problematic due to the importance of a clear theory of change in informing the development of health interventions [104]. While there is evidence to support the beneficial effects of digital interventions in alleviating social isolation, the lack of clear theory-driven approaches and the preliminary nature of many studies prevent definitive conclusions from being made.

Also noteworthy is the lack of interventions specifically designed to alleviate social isolation outcomes, apart from the two studies [65•, 66•] utilizing the +Connect app, which was designed to reduce loneliness among young adults. Most studies used broadly oriented interventions, assessing a number of therapeutic targets related to the mental health conditions of the sample populations involved. In these studies, social isolation was not the primary outcome, but instead it was included among a range of psychosocial outcomes. These findings indicate that the true effect of using technology to alleviate social isolation among individuals with mental health conditions might be masked due to the less-focused designs and broader approaches.

### Implications

This scoping review addresses an important gap in the literature by synthesizing a wide body of evidence assessing digital interventions for social isolation among individuals with mental health conditions. However, there is a lack of research for social isolation interventions using novel technologies (e.g., VR and robotics) and targeting those with mental health problems. While we have found a variety of feasible approaches to be tested in future studies, evidence of the effectiveness of these approaches used in digital interventions was insufficient. The lack of high-quality studies and the lack of theory-driven approaches in the current evidence base limits our ability to make clear recommendations for effective interventions. As such, future research using larger-scale RCTs and assessing interventions with a clear underlying theoretical basis is needed to further explore the potential of these approaches, subject to evidence of the feasibility of those trials.

Furthermore, there is scope to explore the effects of digital interventions on other objective social isolation outcomes, such as social network sizes or social connections for people with mental health problems [105]. Future studies are needed to capture the effectiveness of elements of persuasive technology, including reminders, regular check-ins and tailored content, which have been found to increase the engagement of users. This is a potential way to reduce the high attrition rates consistently found in digital interventions and should be further developed in future research [101, 102]. Based on this review, the existing evidence does not currently support implementing these interventions on a large scale in clinical practice, though studies included in this review provide a starting point for future research in this area.

### Limitations

The methodological limitations identified in the existing evidence should be taken into consideration when reviewing the findings. The major limitations include the lack of fully powered RCTs, studies with clearly specified primary outcome(s) and interventions specifically designed to alleviate social isolation, and prevent definitive conclusions to be made, particularly in relation to clinical effectiveness of the different types of interventions. In addition, several other limitations should be considered when interpreting our findings. First, the exclusion of qualitative studies, grey literature and unpublished studies mean that we might have overlooked findings from other potentially relevant studies that might have allowed us to obtain a more comprehensive view of current evidence in an emerging field, including on experiences of using the interventions. Second, although our decision to include feasibility trials may have allowed us to examine more novel approaches, this introduced greater heterogeneity of studies, limiting the comparability of findings across studies. As expected, due to the variations in study characteristics, meta-analysis was judged unsuitable. Third, despite not using geographical or language restrictions during our search, we did not retrieve eligible articles in languages other than English, although we may have missed some written in other languages. This also limits the generalisability of findings to other cultures as most included trials were conducted in the United States or in Australia.

## Conclusions

Our scoping review identified web-based programmes, phone-based programmes, blended interventions, socially assistive robot and virtual reality as the types of digital intervention used for addressing social isolation in individuals with mental health conditions. The lack of studies of high methodological quality limited our ability to make firm conclusions about their clinical effectiveness. Therefore, this review has identified the need to conduct high-quality studies to improve the evidence base in this area. We also identified a need for research to assess and develop theoretically-driven approaches, as well as interventions that specifically target social isolation for individuals with mental health conditions.

## Supplementary Information

**Additional file 1.** Search terms.

**Additional file 2.** Tables of quality assessments using MMAT.

## Data Availability

All data generated or analysed during this study are included in this published article [and its supplementary information files].

## Abbreviations

ACM: Association of Computing Machinery
BA: Behavioural Activation
BMI: Body Mass Index
CATCH-IT: Competent Adulthood Transition with Cognitive-behavioural and Interpersonal Training
CBT: Cognitive Behavioural Therapy
CES-D: Center for Epidemiological Studies-Depression
CHAT: Connecting to Help After Trauma
CMTR: Chinese version of My Trauma Recovery
DAD: Dorehye Amozeshie Dokhtaran
DSSI: Duke Social Support Index
ESM: Experience Sampling Method
ESRC: Economic and Social Research Council
FoH: Family of Heroes
FSSQ: Functional Social Support Questionnaire
IEEE: Institute of Electrical and Electronics Engineers
INQ: Interpersonal Needs Questionnaire
IPT: Interpersonal Therapy
ISG: Internet Support Group
IVR: Interactive Voice Response
KHL: Kids Helpline
LSNS: Lubben Social Network Scale
MDD: Major Depressive Disorder
MI: Motivational Interviewing
MMAT: Mixed Methods Appraisal Tool
MOS-SSS: Medical Outcomes Trial-Social Support Scale
MOST: Moderated Online Social Therapy
MSPSS: Multidimensional Scale of Perceived Social Support
OSF: Open Science Framework
PACE: Psychiatry Access, Continuity, and Evaluation
Paro: Personal Assistant Robot
PICO: Population, Intervention, Comparison and Outcomes
PSSS: Perceived Social Support Scale
PTSD: Post-Traumatic Stress Disorder
PWS: Person(s) with Schizophrenia
RCT: Randomised controlled trial
SAD: Social Anxiety Disorder
SCS: Social Connectedness Scale
SCT: Social Cognitive Theory
SDT: Self-Determination Theory
SMARTapp: Schizophrenia Mobile Assessment and RealTime feedback application
SNS: Social networking service
SPS: Social Provisions Scale
SSQ: Social Support Questionnaire
TAU: Treatment-as-usual
TT: Touch technology
UCLA-LS: University of California, Los Angeles-Loneliness Scale
UHR: Ultra-high risk
UK: United Kingdom
UKRI: United Kingdom Research and Innovation
USA: United States of America
WEI: Website Evaluation Instrument
VR: Virtual reality

## Footnotes

1 One trial involved the same sample population as another trial [53•, 75•]. Thus, the participants were only counted once. Two trials involved dyads of participants consisting of persons with mental health disorders and their family members/support persons – each individual is counted as individual participants [60, 81].
