# Supplementary material for: Digital interventions for subjective and objective social isolation among individuals with mental health conditions: a scoping review
Source: BMC Psychiatry. 2022 May 12;22:331. doi: 10.1186/s12888-022-03889-0 (PMC9098213; doi:10.1186/s12888-022-03889-0)
Supplement: Supplementary file 1 — Additional file 1. Search terms. [file 12888_2022_3889_MOESM1_ESM.docx]

**Search terms**

Following the PICO framework, the following search terms were used with three databases (MEDLINE, Embase and PsycINFO):

- Population:
  - Mental OR psychiatr* OR schizo* OR psychosis OR psychotic OR depress* OR mania* OR manic OR bipolar OR anxiety OR phobia* OR obsessive-compulsive disorder* OR panic disorder* OR post-traumatic stress disorder* OR eating disorder* OR personality disorder* OR mood disorder*
- Intervention:
  - digital OR web* OR technolog* OR electronic OR internet OR online OR smartphone OR phone OR mobile OR social media OR computer OR tablet OR virtual reality OR augmented reality OR robot* OR user interface* OR voice interface* OR speech interface* OR intelligent assistant* OR voice assistant* OR prototype
  - intervention OR therapy OR treatment
- Comparators: none
- Outcomes:
  - social isolation OR loneliness OR social network* OR social support OR confiding OR confide OR social contact* OR social relation* OR social capital

*Search terms for the outcomes are taken from a conceptual review of social isolation (72). Asterisks (*) are employed as wildcard symbols to broaden the search by including variations of a term.

Slight modifications were made on the search terms used to search IEEE Xplore and ACM digital library due to the limitations on the search engines. However, the semantics of search terms were unchanged:

- Population:
  - Mental OR psychiatr* OR schizo* OR psychosis OR psychotic OR depress* OR mania* OR manic OR bipolar OR anxiety OR phobia OR "obsessive compulsive disorder" OR "panic disorder" OR "post-traumatic stress disorder" OR "eating disorder" OR "personality disorder" OR "mood disorder"
- Intervention:
  - digital OR web* OR technolog* OR electronic OR internet OR online OR smartphone OR phone OR mobile OR "social media" OR computer OR tablet OR "virtual reality" OR "augmented reality" OR robot* OR "user interface" OR "voice interface" OR "speech interface" OR "intelligent assistant" OR "voice assistant" OR prototype
  - intervention OR therapy OR treatment
- Comparators: none
- Outcomes:
  - "social isolation" OR loneliness OR "social network" OR "social support" OR confiding OR confide OR "social contact" OR "social relation" OR "social capital"
