# Supplementary material for: Digital interventions for subjective and objective social isolation among individuals with mental health conditions: a scoping review
Source: BMC Psychiatry. 2022 May 12;22:331. doi: 10.1186/s12888-022-03889-0 (PMC9098213; doi:10.1186/s12888-022-03889-0)
Supplement: Supplementary file 2 — Additional file 2. Tables of quality assessments using MMAT. [file 12888_2022_3889_MOESM2_ESM.docx]

**Tables of quality assessments using MMAT**

Table A: quality assessments of feasibility studies using MMAT

| **Quantitative randomized controlled trial(s) (n=3)** | | | | | | | | |
| --- | --- | --- | --- | --- | --- | --- | --- | --- |
| **Author, year** | **Clear research questions (or objectives)** | **Collected data address the research question** | **Randomization appropriately performed** | **Groups comparable at baseline** | **Complete outcome data** | **Outcome assessors blinded to the intervention provided** | **Participants adhere to the assigned intervention** | **Risk of bias** |
| Rotondi, 2005 | Yes | Yes | Unclear | Unclear | Yes | Unclear | No | High |
| O’Mahen, 2014 | Yes | Yes | Yes | Unclear | No | Unclear | No | High |
| Hanssen, 2020 | Yes | Yes | Unclear | Yes | No | Unclear | No | High |
| **Quantitative non-randomized trial(s) (n=8)** | | | | | | | | |
| **Study first author, year** | **Clear quantitative research questions** | **Collected data address the research question** | **Participants representative of the target population** | **Measures appropriate regarding both the outcome and intervention (or exposure)** | **Complete outcome data** | **Confounders accounted for in the design and analysis** | **Intervention administered (or exposure occurred) as intended** | **Risk of bias** |
| Van Voorhees, 2005 | Yes | Yes | Yes | Yes | Unclear | No | Unclear | Moderate |
| Rice, 2020 | Yes | Yes | Yes | Yes | Yes | No | Yes | Low |
| Rice, 2018 | Yes | Yes | Yes | Yes | Yes | No | Yes | Low |
| Price, 2014 | Yes | Yes | No | Yes | No | No | Unclear | High |
| Pfeiffer, 2016 | Yes | Yes | Yes | Unclear | No | No | Yes | Moderate |
| Ludwig, 2020 | Yes | Yes | Yes | Yes | Yes | No | Yes | Low |
| Bailey, 2020 | Yes | Yes | Yes | Yes | Yes | No | Unclear | Moderate |
| Alvarez-Jimenez, 2018 | Yes | Yes | Yes | Yes | Yes | No | Yes | Low |
| **Mixed-methods study(s) (n=5)** | | | | | | | | |
| **Study first author, year of publication** | **Clear research questions (or objectives)** | **Collected data address the research question** | **Adequate rationale for using mixed methods** | **Different components effectively integrated** | **Outputs of integration adequately interpreted** | **Divergences and inconsistences adequately addressed** | **Different components adhere to quality criteria of each method involved** | **Comments** |
| Lim, 2019 | Yes | Yes | Yes | Yes | Yes | No | Yes | Low |
| Lim, 2020 | Yes | Yes | Yes | Yes | No | Yes | No | Moderate |
| Gjerdingen, 2013 | Yes | Yes | Yes | Yes | No | No | No | High |
| Dow, 2008 | Yes | Yes | Yes | Unclear | Yes | Yes | No | Moderate |
| Campbell, 2019 | Yes | Yes | Yes | Yes | No | Yes | No | Moderate |

Table B: quality assessments of single-group studies, using MMAT

| **Quantitative non-randomized trials (n=4)** | | | | | | | | |
| --- | --- | --- | --- | --- | --- | --- | --- | --- |
| **Study first author, year** | **Clear research questions (or objectives)** | **Collected data address the research question** | **Participants representative of the target population** | **Measures appropriate regarding both the outcome and intervention (or exposure)** | **Complete outcome data** | **Confounders accounted for in the design and analysis** | **Intervention administered (or exposure occurred) as intended** | **Risk of bias** |
| Wang, 2016 | Yes | Yes | Yes | Yes | Yes | Yes | Unclear | Low |
| Lee, 2018 | Yes | Yes | Yes | Yes | Unclear | Yes | Unclear | Moderate |
| Goodwin, 2018 | Yes | Yes | Yes | Yes | No | No | Unclear | Moderate |
| De Almeida, 2018 | Yes | Yes | No | Yes | Yes | No | Yes | Moderate |
| **Mixed-methods study(s) (n=3)** | | | | | | | | |
| **Study first author, year of publication** | **Clear research questions (or objectives)** | **Collected data address the research question** | **Adequate rationale for using mixed methods** | **Different components effectively integrated** | **Outputs of integration adequately interpreted** | **Divergences and inconsistences adequately addressed** | **Different components adhere to quality criteria of each method involved** | **Risk of bias** |
| Loi, 2016 | Yes | Yes | No | Unclear | No | Yes | No | High |
| Chen, 2020 | Yes | Yes | No | No | Unclear | Yes | Yes | Moderate |
| Aschbrenner, 2016 | Yes | Yes | Yes | Yes | Yes | Yes | No | Low |

Table C: quality assessments of effectiveness trials using MMAT

| **Quantitative randomized trials (n=8)** | | | | | | | | |
| --- | --- | --- | --- | --- | --- | --- | --- | --- |
| **Author, year** | **Clear research questions (or objectives)** | **Collected data address the research question** | **Randomization appropriately performed** | **Groups comparable at baseline** | **Complete outcome data** | **Outcome assessors blinded to the intervention provided** | **Participants adhere to the assigned intervention** | **Risk of bias** |
| Van Voorhees, 2008 | Yes | Yes | Yes | Yes | Yes | No | No | Moderate |
| Saulsberry, 2013 | Yes | Yes | Yes | Yes | No | Yes | No | Moderate |
| Pot-Kolder, 2018 | Yes | Yes | Yes | Yes | Yes | Yes | Yes | Low |
| Moeini, 2019 | Yes | Yes | Unclear | Yes | No | Unclear | Unclear | High |
| Marasinghe, 2012 | Yes | Yes | Unclear | Yes | Yes | Unclear | Yes | Moderate |
| Kaplan, 2014 | Yes | Yes | Yes | Yes | No | Unclear | Unclear | Moderate |
| Kaplan, 2011 | Yes | Yes | Yes | Yes | Yes | Unclear | No | Moderate |
| Interian, 2016 | Yes | Yes | Yes | Yes | Yes | Unclear | Unclear | Moderate |
| **Mixed-methods trials (n=1)** | | | | | | | | |
| **Study first author, year of publication** | **Clear research questions (or objectives)** | **Collected data address the research question** | **Adequate rationale for using mixed methods** | **Different components effectively integrated** | **Outputs of integration adequately interpreted** | **Divergences and inconsistences adequately addressed** | **Different components adhere to quality criteria of each method involved** | **Risk of bias** |
| Ellis, 2011 | Yes | Yes | Yes | Yes | Yes | No | No | Moderate |
